# A Unique THN Motif Is Critical for Enabling Efficient C‐Terminal Traceless Cleavage

**DOI:** 10.1002/advs.202501991

**Published:** 2025-04-07

**Authors:** Ruocheng Gu, Yunuo Lin, Rouyu Di, Tongtong Zhou, Tingwen Fan, Wei Li, Lili Miao, Huaiyi Yang

**Affiliations:** ^1^ Department of Microbial Physiological & Metabolic Engineering State Key Laboratory of Microbial Diversity and Innovative Utilization Institute of Microbiology Chinese Academy of Sciences Beijing 100101 China; ^2^ University of Chinese Academy of Sciences Beijing 100049 China; ^3^ Beijing Key Laboratory of Genetic Element Biosourcing & Intelligent Design for Biomanufacturing Beijing 100101 China

**Keywords:** gp41‐1, high cleavage activity, traceless cleavage, THN motif

## Abstract

Traceless protein cleavage is a significant challenge in intein application, as most common inteins studied today are not both active and promiscuous. In this study, the intein gp41‐1 is engineered, which demonstrates the most efficient traceless cleavage reported to date and shows high compatibility to 1st amino acid. The evidence provided for the first time is that the unique THN motif, which is prevalent in class 3 inteins, is essential for achieving high‐efficiency traceless C‐terminal cleavage. The hydrogen bond between the hydroxyl group of Thr_123_ and the main chain of His_124_ is suggested to be indispensable for stabilizing the THN motif to separate Asp_107_ (the limiting factor for C‐cleavage) from Asn_125_ and the C‐extein residues from the active sites, which jointly lead to the highest traceless C‐cleavage activity. Both cleavage data and molecular dynamics (MD) simulations results demonstrate that mutating Thr_123_ greatly disturbed the THN motif, leading to inactivity. These findings reveal a pivotal motif for intein traceless cleavage efficiency, providing valuable insights for designing inteins with enhanced traceless C‐terminal cleavage capabilities in future applications.

## Graphical abstract

THN is identified as the key motif to facilitate the rapid traceless cleavage of gp41‐1. The deflection of THN motif not only avoids the interaction between C‐exteins and active sites, but also separates Asp_107_ away from Asn_125_. In addition, a flexible block F contributes to faster C‐cleavage. The unique THN offers a new insight for identifying high active inteins.

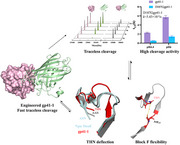

## 1 Introduction

**Figure 1:**
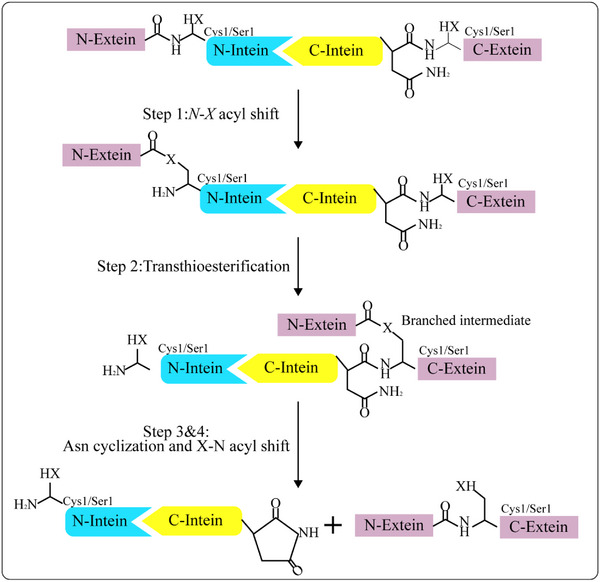
Mechanism of protein trans‐splicing (PTS). 1) An intermediate in which the sulfhydryl or alcohol group of the first N‐terminal amino acid (Cys/Ser) attacks the peptide bond at N‐extein/intein junction to form a thioester or oxoester; 2) The intermediate of the N‐terminal thioester or oxoester is transferred to the side chain of the Cys +1 of the C‐extein; 3) The cyclization of the highly conserved Asn at the C terminus of the intein forms a succinimide and releases the intein; 4) the thioester or oxoester bond between the exteins rearranges to a peptide bond by a spontaneous S─N or O─N acyl shift. In general, X can be sulfur or oxygen.

Inteins are sequences within long proteins that can catalyze their own removal from the protein and splice both flanking fragments (the N‐extein and C‐extein) to form a complete protein.^[^ ^1^ ^]^ The first intein was identified from the vacuolar proton‐translocating ATPase gene of *Saccharomyces cerevisiae* (Sce VMA1) in 1990.^[^ ^2^ ^]^ Since then, multiple inteins have been discovered, and their mechanism is highly conserved, occurring by the following four steps: 1) N‐S/O acyl shift, 2) transesterification, 3) succinimide formation, and (4) S/O‐N acyl shift^[^ ^3^ ^]^ (**Figure** **1**). Inteins have been extensively applied in various biotechnological applications, including protein‒protein interactions,^[^ ^4^ ^]^ and for gene therapy,^[^ ^5^ ^]^ biosensors^[^ ^6^ ^]^ and especially protein purification applications.^[^ ^7^ ^]^ However, achieving efficient and traceless cleavage for protein purification remains challenging.

Intein sequences share a low homology, but they still have several conserved residues, such as His residues, the first residue at the N‐terminus (which is Cys or Ser) and the last residue at the C‐terminus (Asn).^[^ ^8^ ^]^ N‐ or C‐terminal cleavage activity is inhibited if the Cys/Ser or Asn/Gln residues are mutated to Ala.^[^ ^9^ ^]^ Considering their C‐ or N‐cleavage properties, inteins have been applied to purify exogenous proteins of interest via traceless tag removal.^[^ ^10^ ^]^ However, efficient and traceless cleavage to purify exogenous proteins is difficult to achieve due to several inherent factors. a) The most important factor is that N‐ or C‐cleavage is actually a side reaction that occurs with low efficiency compared with the evolutionarily designed function of naturally occurring inteins, which is protein splicing. b) Many reports have confirmed that retaining the intein domain and the pivotal first C‐extein residue is crucial for splicing, and the splicing process is disturbed once mutations occur in the extein residues. For example, in Npu DnaE, mutation of the Cys+1 residue led to the slow formation of a branched thioester intermediate.^[^ ^11^ ^]^ c) Inteins are naturally located between highly conserved residues in numerous different endogenous host proteins due to evolutionary pressure, and they display context‐dependent activity, especially in terms of the sequences at their splice junctions. For example, the intein Npu DnaE showed little tolerance for variations in the N‐extein residues,^[^ ^12^ ^]^ whereas C‐terminal cleavage was abolished when the tripeptide CFN. However, in practical applications, it is desirable to obtain an intein that could perform complete traceless cleavage with higher cleavage activity.

Several artificial inteins have been designed with the aim of increasing cleavage activity and non‐native residue tolerance, e. g. the DnaE intein family, which includes Ssp DnaE derived from *Synechocystis* species PCC6803^[^ ^13^ ^]^ and Npu DnaE from *Nostoc punctiforme*.^[^ ^14^ ^]^ Wood et al introduced a single mutation D422G to mini‐MtuRecA, which increased the C‐terminal cleavage activity.^[^ ^15^ ^]^ Moreover, engineered Npu DnaE by mutating Asp_118_ to Gly_118_ at the C‐terminus, which increased the C‐terminal cleavage activity to reach 80% within 3 h at room temperature.^[^ ^16^ ^]^ However, these widely used inteins still face a common problem, its poor tolerance to non‐native extein residues. Thus, the essential nature of native extein residues limits the further application of inteins in the production of proteins that do not contain extra residues. Although several reports have shown that intein mutations could enhance this tolerance to a certain extent,^[^ ^17^ ^]^ it remains necessary to construct a highly active intein without non‐native residues. The intein gp41‐1 is the smallest intein (125 aa) and has a weak effect on the folding of the target protein. gp41‐1 is known to perform the fastest C‐terminal cleavage with its native C‐extein residues SSSGV, but its cleavage activity is altered in the absence of this sequence.^[^ ^18^ ^]^ However, these properties provide an entry point for exploring the potential of gp41‐1 as an intein with high tolerance for C‐extein residues.

In this study, a gp41‐1 mutant was designed rationally on the basis of sequence alignment and crystal structure data, and the engineered gp41‐1 could perform completely traceless cleavage with increased activity. We identified that the THN motif of gp41‐1 was crucial to obtain the highest C‐cleavage activity. The results of the cleavage assay proved that the hydroxyl group of Thr_123_ contributed to observed rapid cleavage, and both the cleavage activity and promiscuity further increased with the D107G mutant. Structural analysis and molecular dynamics (MD) simulations further revealed that the interaction between Thr_123_ and His_124_ led to rotation of the THN motif, which not only separated Asp_107_ from Asn_125_ but also increased the distance between the C+1 residue and the active site. Thus, gp41‐1 can be used to perform traceless cleavage at the fastest rate to date. Overall, this work might provide a target for identifying a group of inteins that can perform traceless cleavage and lead to the discovery of novel intein classes. Moreover, the engineered gp41‐1 with high C‐cleavage activity has great potential for protein preparation at both the laboratory and industrial scales.

## 2 Results and Discussion

### 2.1 gp41‐1 Presented the Highest Tolerance for C‐Extein Residues and Traceless Cleavage Activity

**Figure 2:**
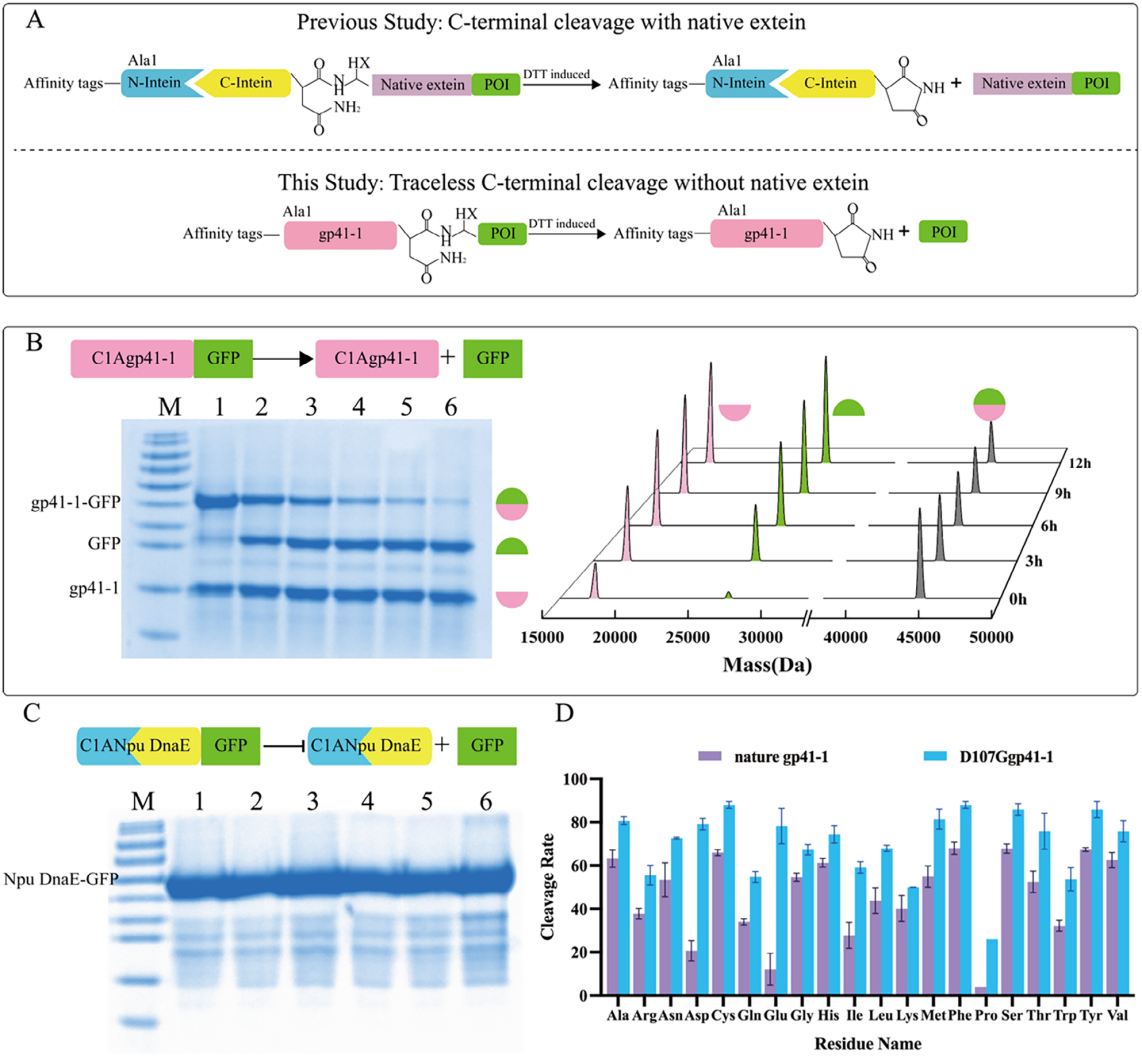
Traceless cleavage activity of gp41‐1 and the D107Ggp41‐1 mutant. A) Schematic of traceless C‐cleavage by gp41‐1. The other inteins evaluated could perform C‐cleavage with only in the presence of the native extein. B) SDS‐PAGE analysis of the cleavage products of gp41‐1‐GFP, which conducted traceless cleavage, LC‐MS detection of the cleavage products at different time points. gp41‐1 produced cleavage products presented similar molecular weights to their standard molecular weights (gp41‐1: 17.5 kDa; GFP: 26.78 kDa; gp41‐1‐GFP: 44.3 kDa). C) Npu DnaE showed no cleavage activity without CFN introduced. D) Saturation mutation of the first residue of GFP to investigate the C+1 residue tolerance of both gp41‐1 and the D107Ggp41‐1 mutant (n = 3). The D107G mutant presented better promiscuity (only Pro was not tolerated), whereas gp41‐1 was unable to perform efficient cleavage when the C+1 residue was Asp or Glu.

The intein gp41‐1 is the fastest and smallest intein identified to date, and the intein Npu DnaE is a typical intein that has been well studied. To benchmark the performance of traceless cleavage of gp41‐1, its traceless cleavage efficiency was compared with that of the well‐characterized Npu DnaE intein. Mutation Cys_1_ to Ala_1_ could inhibit N‐terminal cleavage activity, therefore gp41‐1 (C1A) and Npu DnaE (C1A) were fused to GFP without the introduction of native junction residues, respectively. Unlike gp41‐1, which achieved traceless cleavage without native extein residues (**Figure** **2**B), Npu DnaE strictly required its native extein CFN for activity (Figure 2C; Figure S1, Supporting Information), underscoring the superior versatility of gp41‐1. Even though artificial Npu DnaE showed a higher cleavage activity than gp41‐1, yet the requirement of native exteins made it unsuitable for producing functional proteins (**Table** **1**). Some C1A inteins may still be able to perform protein splicing;^[^ ^16^ ^]^ thus, a plasmid was constructed to produce Trx‐gp41‐1‐GFP, and the splicing product Trx‐GFP was not produced during tag removal (Figure S2, Supporting Information). Detection of GFP on the basis of its calculated molecular weight further proved that gp41‐1 could perform traceless cleavage (Figure 2B; Figure S3, Supporting Information). However, the traceless cleavage activity of gp41‐1 was lower than gp41‐1 with its native exteins attached.

**Table 1:** The C‐terminal cleavage efficiency of gp41‐1 and other inteins.

Intein	C‐terminal cleavage (t_1/2_)	Protein of interest	Advantage	Drawback	Refs.
Npu DnaE (CFN)	37 °C, 16 min	GFP	High efficiency	Poor tolerance for non‐native residues.	[16]
Ssp DnaE (CFN)	23 °C, 1 h	Trx			[22]
gp41‐1 (SSSGV)	37 °C, 5 min	Trx			[18]
IMPACT	37 °C, > 16 h	Cph2	Residue tolerant	Low cleavage activity.	[23]
gp41‐1(this work)	37 °C, 3 h	GFP	Traceless cleavage	Low efficiency.	
D107Ggp41‐1 (this work)	37 °C, 30 min	GFP	Traceless cleavage, high efficiency.		

Even though C‐terminal cleavage is a relatively independent process, it is strongly influenced by the C‐terminal residues. Saturation mutation of the first amino acid of GFP was subsequently conducted to investigate the C+1 tolerance of gp41‐1. Similar to that observed with Ssp DnaB^[^ ^19^ ^]^ and Npu DnaE,^[^ ^20^ ^]^ changing the C+1 residue affected the cleavage activity of gp41‐1(Figure 2D). gp41‐1 presented good tolerance to a variety of C+1 residues, with the exceptions of Asp, Glu and Pro. Based on sequence and structural analysis, a single point mutation (D107G) was introduced in gp41‐1. Cleavage assay revealed that the D107G mutant presented the best performance, with increased promiscuity and cleavage activity (Figure 2D). The rate constant (*k*) for the D107G mutant reached 1.2 × 10^−4^ s^−1^, which was tenfold greater than that of natural gp41‐1 (≈10^−5^ s^−1^) (Figure 4B). Moreover, the rate constant of the D107G mutant increased further to 5.43×10^−4^ s^−1^ in the presence of sodium acetate, which was five times greater than that of Tris buffer (pH 6) and approximately the same level as that of gp41‐1 with its native residues SSSGV. Similar cleavage data were obtained with other target proteins, including MBP and GST (Figure 4E). Even though plenty of inteins and their artificial counterparts have been applied in protein purification. However, these well‐studied inteins are limited in that they do not exhibit both high C‐cleavage activity and promiscuity (Table 1). Compared with other reported inteins, D107Ggp41‐1 cleavage is highly efficient. The engineered gp41‐1 achieved 50% cleavage in 30 min at 37 °C without the inclusion of any native exteins, whereas other well‐studied inteins, such as those in the DnaE family, can perform cleavage only in the presence of the native C‐extein residues (Figure S1, Supporting Information).^[^ ^21^ ^]^ Notably, since expressing gp41‐1 as a continuous intein, unexpected cleavage caused a loss of target proteins. However, gp41‐1 presented a lower unexpected cleavage (<40%) in vivo. In addition, unexpected cleavage decreased to 20% under 16 °C cultivation. So, it could be alleviated by changing culture condition (Figure S4, Supporting Information). These data indicated that gp41‐1 has great potential for protein purification and tag removal applications.

### 2.2 The Unique THN Motif of gp41‐1 Led to a Greater Deflection

**Figure 3:**
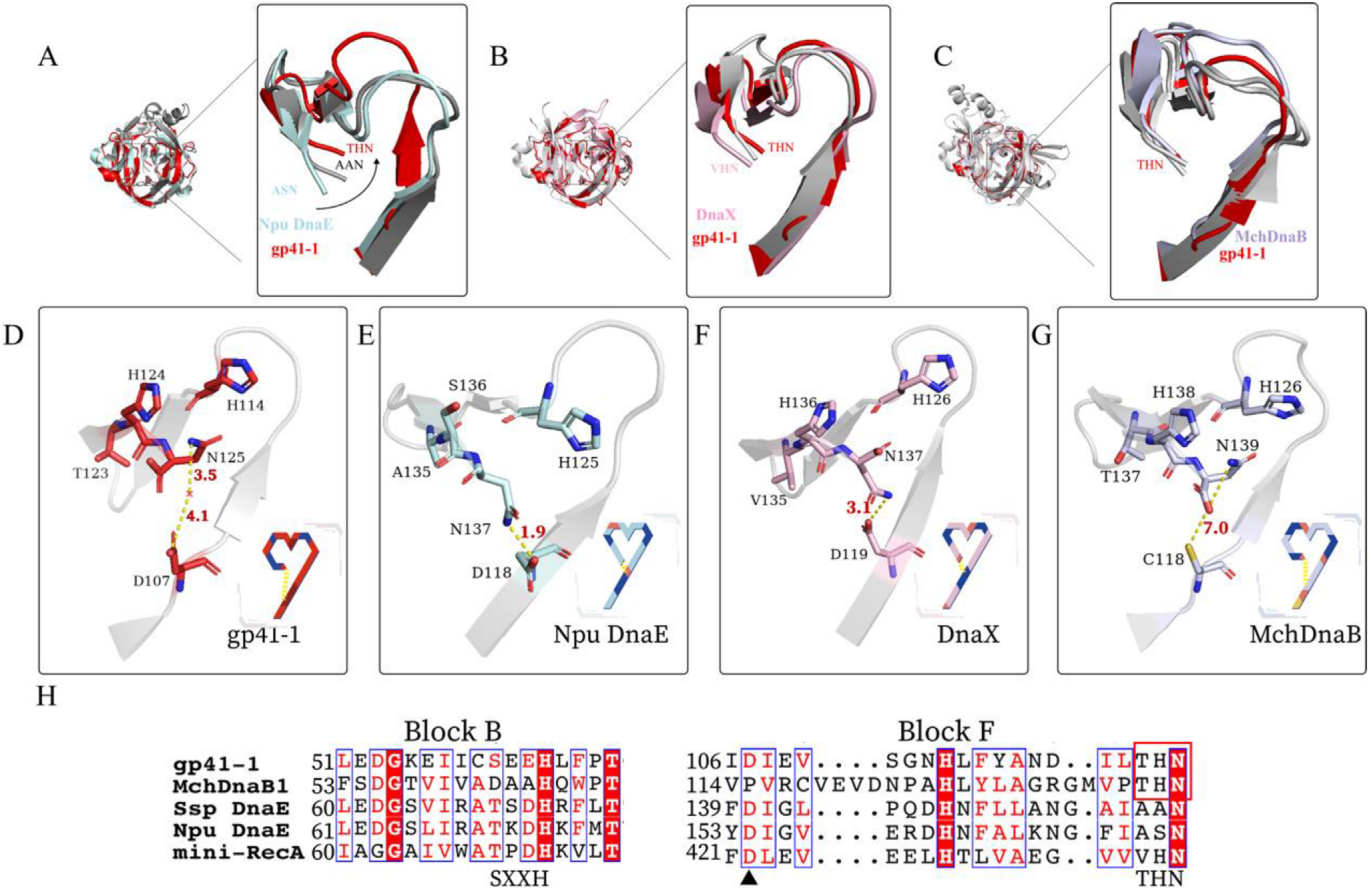
Comparison of the THN motif of gp41‐1 with those of other inteins. A) The structure of the gp41‐1 THN motif was compared to the AAN/ASN motifs of Npu DnaE and Ssp DnaE, and an obvious deflection of the THN motif of gp41‐1 was observed. B) Alignment of the gp41‐1 THN motif with the LHN/VHN motifs of the inteins DnaX and prp8. Deflection of the terminal tripeptide was observed with all of these inteins, yet the deflection of gp41‐1 was the greatest. C) Inteins with a THN or SHN motif presented deflection similar to that of gp41‐1, and the Asp and Asn residues were also far apart. D–G) Such a deflection led a change of distance between Asp and Asn. H) Sequence alignment showed that gp41‐1 presented a different block B and THN motif that was similar to class 3 inteins.

To investigate the mechanism by which gp41‐1 performs rapid, traceless cleavage, sequence alignment of gp41‐1 and members of the DnaE split intein family (class 1) was performed, including Npu DnaE, Ssp DnaE, and MtuRecA, and a class 3 intein (Figure S5A, Supporting Information), and revealed several interesting characteristics. a) An Asp in block F of gp41‐1 is conserved among class 1 inteins, especially those in the DnaE intein family, whereas this residue is usually Cys in class 3 inteins.^[^ ^24^ ^]^ The conserved Asp in block F has been reported as a rate‐limiting factor for C‐terminal cleavage; b) Block B of the DnaE split intein family is a conserved TXXH motif,^[^ ^25^ ^]^ whereas the sequence of block B is SXXH in gp41‐1 and DXXH in class 3 inteins (Figure 3H; Figure S5A, Supporting Information). The His in block B has been assumed to be involved in N‐S/O acyl shift, transesterification, and the interplay between the intein and extein sequences.^[^ ^26^ ^]^ The unusual block B of gp41‐1 might be responsible for its rapid splicing reaction. However, splicing mechanism was not considered in this paper. c) The terminal tripeptide segment of gp41‐1 and class 3 inteins is Thr‐His‐Asn (THN). The unique Thr residue in gp41‐1 is conserved among class 3 inteins, whereas it is sporadically distributed in class 1 inteins. Most Eubacteria and Archaea inteins are associated with DNA synthesis, while in viruses and phages, only 17% of inteins are involved in such processes.^[^ ^27^ ^]^ Moreover, class 1 inteins have been linked to DNA synthesis, whereas more class 3 inteins are involved in ATP synthesis.^[^ ^28^ ^]^ Block F is similar in intein gp41‐1 and class 1 inteins, whereas block B in gp41‐1 and class 3 inteins are similar. Thus, these results suggested that gp41‐1 represents a transition evolution between class 1 and class 3 inteins.

The structure of gp41‐1 showed the ten residues that surrounded Asn_125_ (<5 Å) were His_63_, Asp_107_, Ile_108_, Glu_109_, Val_110_, His_114_, Leu_115_, Phe_116_, Thr_123_, and His_124_. These residues are highly conserved in most inteins, except Thr_123_. Among them, His_114_ and His_124_ are active centres that are involved in the cleavage process.^[^ ^29^ ^]^ The active centre structures of gp41‐1 was further compared to most other reported inteins.^[^ ^30^ ^]^ The crystal structure of gp41‐1 was very different from that of typical class 1 inteins and much more similar to that of class 3 inteins (**Figure** **3**A,C). Owing to the unique Thr residue in gp41‐1, we focused on the structure of the terminal tripeptide motif (THN) of gp41‐1 and the ASN motif of Npu DnaE (Figure S5B, Supporting Information). There was an obvious deflection of the THN motif in gp41‐1, which may be due to this unique Thr residue. We then extensively compared the terminal tripeptide of gp41‐1 with those of other terminal inteins (Figure 3B). The inteins that displayed motion of the X‐His‐Asn motif also exhibited deflection, but to a lesser degree that that observe in the gp41‐1 structure.^[^ ^31^ ^]^ Overall, the structures of gp41‐1 and class 3 inteins are similar,^[^ ^28^, ^32^ ^]^ especially in terms of the deflection of the THN sequence. Because of its similarity to class 3 inteins, gp41‐1 was designed to work as a class 3 intein in a previous study; however, this attempt failed.^[^ ^33^ ^]^ Additionally, similar deflections were observed with other inteins containing the terminal tripeptide Ser‐His‐Asn,^[^ ^34^ ^]^ which suggested that the critical Thr or Ser residue was responsible for the observed greater deflection. And inteins with THN/SHN motif might be an evolution symbol that for a rapid splicing and cleavage process. It requires more evidence to clarify the evolution class of these THN/SHN inteins.

### 2.3 The Hydrogen Bond between the Side Chains of Thr_123_ and His_124_ is Indispensable for Traceless, Rapid C‐Terminal Cleavage

**Figure 4:**
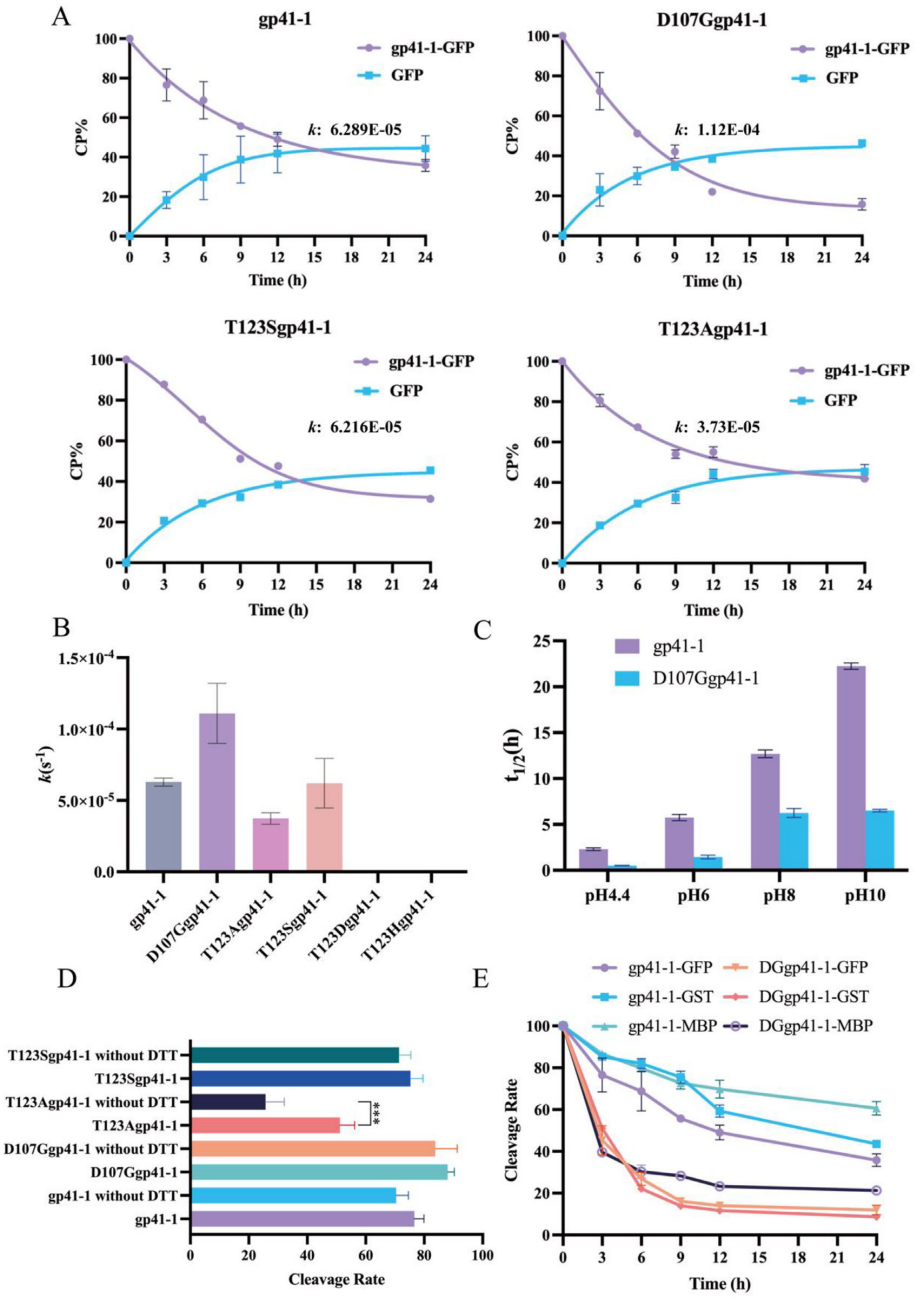
Traceless C‐cleavage activities of gp41‐1 and its mutants. A) Traceless C‐cleavage activities of gp41‐1 and its mutants fused to GFP. gp41‐1 and the T123S mutant had similar cleavage rates, whereas the cleavage rate of the D107G mutant increased and that of the T123A mutant strongly decreased. B) The rate constants (*k* values) for the cleavage reactions of gp41‐1 and its mutants were calculated. gp41‐1 and the T123S mutant had k values of ≈6 × 10^−5^ s^−1^, the *k* value for the T123A mutant decreased to 3 × 10^−5^ s^−1^, while that of the D107G mutant increased to 1 × 10^−4^ s^−1^. C) The pH of the cleavage buffer affected cleavage activity, with lower pH values resulting in higher activity. D) The cleavage activity of gp41‐1 and its mutants were determined in vitro in the absence of DTT (n = 5), and the loss of the hydroxyl group from a Thr or Ser residue strongly inhibited C‐cleavage, p < 0.01. E) The proteins GST and MBP were also fused to gp41‐1 and its D107G mutant, and the cleavage activities were similar to that of gp41‐1‐GFP.

The greater deflection of the THN motif caused by Thr_123_ might be important for C‐terminal cleavage. We hence mutated Thr_123_ to other amino acids (His, Ser, Ala and Asp), but these mutations led to gp41‐1 displaying decreased cleavage activity or no activity (**Figure** **4**A), except for the T123S mutant. Notably, the T123A mutant maintained half activity, whereas the cleavage activities of the T123H and T123D mutants were almost completely abolished (Figure 4B; Figure S6, Supporting Information). The T123A mutant could not establish the important hydrogen bond, and its cleavage activity decreased upon fusion to different C+1 residues (Figure S7, Supporting Information). The cleavage data highlighted the importance of Thr_123_ for traceless cleavage activity, suggesting that the hydroxyl group of Thr_123_ is responsible for the observed traceless, rapid C‐terminal cleavage. An increase in the distance between Asp in block F and Asn (> 7 Å) at the C‐terminus was also found in predicted structures of other inteins (> 10) with THN/SHN motifs in metagenome data (Figure S8, Supporting Information).^[^ ^35^ ^]^ However, little evidence has indicated that this increased distance is beneficial for C‐cleavage. In addition, we performed in vitro cleavage reactions without DL‐dithiothreitol (DTT). The cleavage activity of the Thr_123_ mutants decreased significantly decreased in the absence of DTT, except for T123S (Figure 4D; Figure S9, Supporting Information), whereas the mutants containing the residue Thr_123_ showed similar cleavage activity in both the absence and presence of DTT. This result indicated that the hydroxyl group of Thr_123_ facilitates the cleavage reaction, and not needing the reducing agent DTT greatly lowers downstream costs. In addition, absence of DTT allowed gp41‐1 purification via Ni^+^ column chromatography, whereas other inteins need to be fused to larger fusion tags, such as MBP or GST.^[^ ^36^ ^]^ The crystal structure of gp41‐1 revealed that Thr_123_ forms hydrogen bonds with His_63_ or His_124_. Even though the T123H and T123D mutants were able to form a hydrogen bond with His_63_, their cleavage activity was relatively low. This finding implied that there may actually be no interaction between His_63_ and Thr_123_ exist. Generation of the H63A mutant proved our hypothesis, since the activities of this mutant were not significantly different from that of gp41‐1 (Figure S10, Supporting Information). Our data suggested that while Thr_123_ is important for the Asn‐mediated cyclization of gp41‐1, other unidentified residues must also contribute to catalysis in this step.

gp41‐1 not only has the highest C‐terminal cleavage activity but also the highest splicing activity reported to date. We only revealed that Thr_123_ was crucial for C‐terminal cleavage; however, we did not clarify the mechanism of the high splicing activity of gp41‐1. In addition to the THN motif, the SXXH motif in block B was different from that of classic inteins. We suspected that the unusual block B of gp41‐1 might be responsible for its rapid splicing reaction. Splicing activity is determined by both N‐terminal cleavage and C‐terminal cleavage. We hypothesized that inteins can have either low or high splicing activity depending on the target protein. Taken together, these findings suggest that inteins possessing both SXXH and THN/SHN motifs could have diverged into a new group with high C‐terminal cleavage activity and splicing activities during evolution. We will focus on identifying inteins with such activities in our future work.

### 2.4 Deflection of the THN Motif caused by Thr_123_ Enhanced the Cleavage Activity of gp41‐1 by Increasing the Flexibility of Block F

Deflection of the terminal tripeptide THN increases the distance between Asp_107_ and Asn_125_ (> 7 Å), and a water molecule was present within the hydrogen bonding distance between Asp_107_ and Asn_125_ (Figure 3D). In contrast, Asp_107_ and Asn_125_ of Npu DnaE and Ssp DnaE interacted directly, with distances of 1.9 Å (Figure 3E) and 2.8 Å, respectively.^[^ ^30a^ ^]^ This hydrogen bond between Asp_107_ and Asn_125_ was proven to be a limiting factor for C‐terminal cleavage, which slowed the C‐cleavage rate to favour N‐terminal cleavage.^[^ ^29^, ^37^ ^]^ Furthermore, the distance between the Asp and Asn residues of DnaX (with a VHN motif) was just over 3.2 Å (Figure 3F), whereas this distance in inteins with a THN motif are greater than 7 Å (Figure 3D; Figure S8, Supporting Information). The greater deflection of the THN motif leads to a greater distance between Asp_107_ and Asn_125_, resulting in a more flexible block F (a loop in block F of gp41‐1),^[^ ^30d^ ^]^ whereas block F adopts a complete β‐sheet structure in other inteins. We therefore hypothesized that a flexible block F would facilitate C‐cleavage. Similar to what has been reported with other synthetic inteins, the Asp to Gly mutation further increased the cleavage activity. The rate constant of D107Ggp41‐1 was approximately the same as that of Npu DnaE in the presence of the CFN tripeptide.^[^ ^12^ ^]^ Such increased cleavage activity highlights that a more flexible structure is beneficial for C‐cleavage. The D107G mutant presented significantly increased cleavage activity regardless of which amino acid was in the first position. Compared with gp41‐1 and the other mutants, the GFP‐fused D107G mutants with an N‐terminal Asp or Glu showed the greatest increases in activity, ranging from 20% to 80% (Figure 2D). Moreover, the cleavage activity of the mutant increased significantly after GST and MBP fusion, with activity similar to that of GST‐ and MBP‐fused gp41‐1 (Figure 4E; Figure S11, Supporting Information), and presented better cleavage performance than that with GFP fusion. The consistent cleavage activity of D107Ggp41‐1 upon fusion with different proteins suggested that the D107Ggp41‐1 mutant was the suitable for most protein production at the laboratory and industrial scales. Moreover, C‐cleavage activity was inhibited when the larger, more sterically hindered residue Lys was introduced into the gp41‐1 sequence (Figure S12A, Supporting Information), further proving that the flexibility of block F was beneficial for C‐cleavage.

### 2.5 Deleterious Mutation Led to a Disturbed the THN Motif

**Figure 5:**
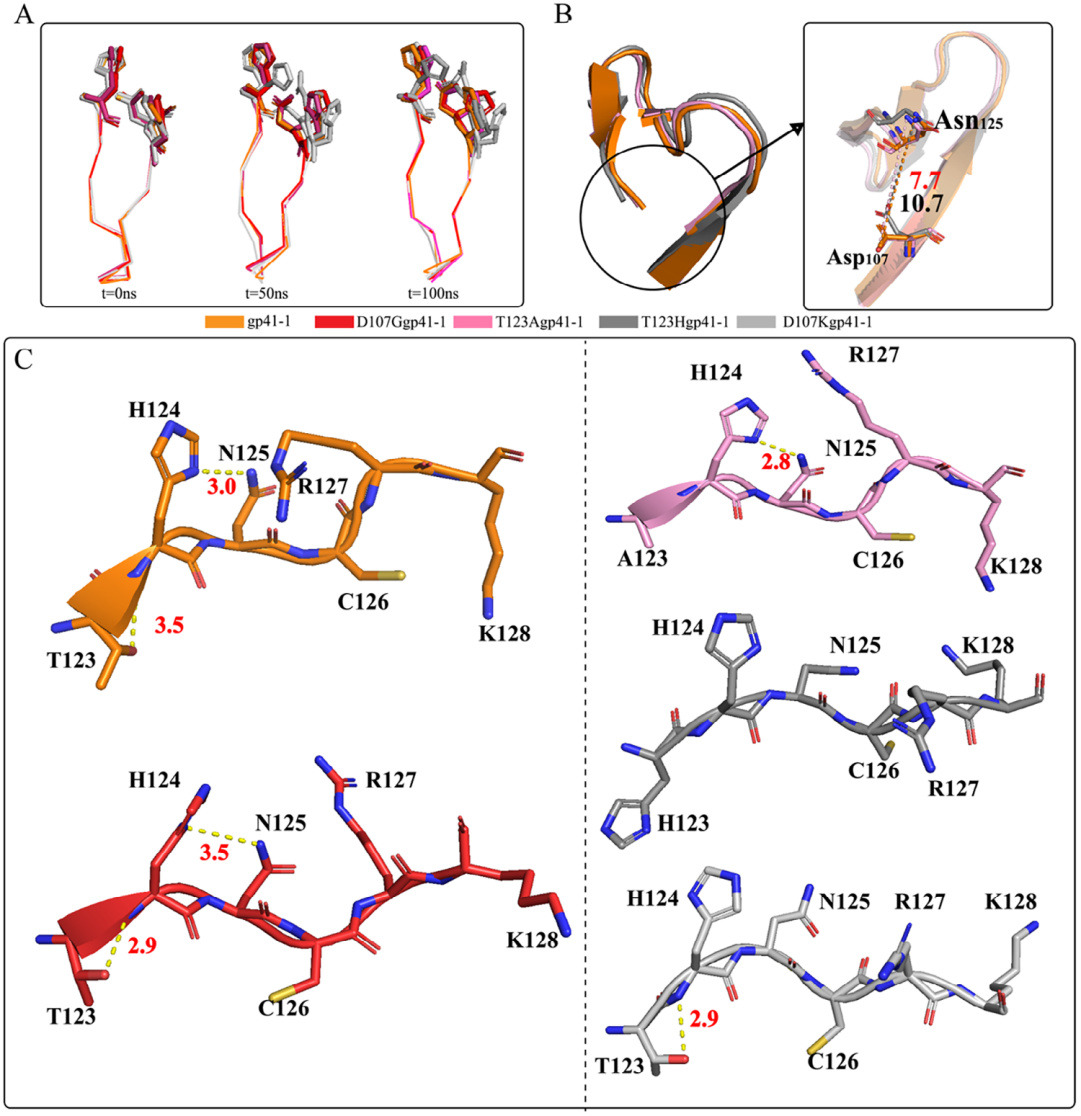
Structures and MD simulations of gp41‐1 and its mutants. A) Structural overlay of residues 114–125 from gp41‐1 (orange), D107Ggp41‐1 (red), T123Agp41‐1 (light pink), D107Kgp41‐1 and T123Hgp41‐1 (grey) after 0, 50, and 100 ns of MD simulations. gp41‐1, D107Ggp41‐1 and T123Agp41‐1 presented similar structures with slight differences in their THN motifs, while the structures of the D107K and T123H mutants were disrupted. B) The distance between Asp_107_ and Asn_125_ in gp41‐1 (orange), T123Agp41‐1 (pink) and T123Hgp41‐1 (grey), His123 mutant showed a closer distance. C) The interactions within THN motif were detected in gp41‐1 and D107G mutant, while these interactions were diminished in inactive mutants. The deflection of THN avoided the interaction between C‐extein and active sites of gp41‐1.

The hydroxyl group of Thr_123_ was involved in the C‐cleavage process. To better understand the interplay between Asp_107_, Thr_123_ and the gp41‐1 active sites, we carried out MD simulations with gp41‐1 and its T123A and D107G mutants. These proteins presented diverse trajectories, especially for the THN motif. gp41‐1 (orange), the D107G mutant (red) and the T123A mutant (white) had compact THN motifs, whereas the non‐active mutants presented much more open THN motifs (**Figure** **5**A). Notably, D107Ggp41‐1 presented the most flexible block F and THN motif among the three active inteins (Figure 5C; Figure S13, Supporting Information). Furthermore, the results suggested that a more flexible structure would be beneficial for cleavage activity. We then noted that the imidazole ring of His_124_ of the D107G mutant adopted a variety of rotated conformations (Figure 5C). After calculating the χ2 angles, and the D107G mutant presented a greater ψ and smaller φ for Asn_125_ than those for gp41‐1 (**Table** **2**). These data indicated that the changes in χ2 angles in the D107G mutant were responsible for increasing the flexibility of the THN motif, which was beneficial for the rapid interaction between His_124_ and Asn_125_.

**Table 2:** The His_124_ χ2 angles calculated from individual MD simulation trajectories for gp41‐1 and its mutants after 100 ns.

Intein	CCN	His_124_(φ)	His_124_(ψ)	Asn_125_(φ)	Asn_125_(ψ)
gp41‐1	39.6	−102.5	158.8	−70.2	164.4
D107Ggp41‐1	42.4	−119.5	156.8	−64.8	177.1
D107Kgp41‐1	57.8	−93.5	161.6	−47.3	141.4
T123Agp41‐1	37.1	−103.5	153	−72.8	174.8
T123Hgp41‐1	29.1	−95.5	167.1	−85.1	149.8
gp41‐1‐GST	15.8	−114.1	130.9	−113	94
D107Ggp41‐1‐GST	30	−82	158	−44	154.8
gp41‐1‐MBP	28.6	−149	−165.9	−147.5	125.1
D107Ggp41‐1‐MBP	36	−108.2	154.4	−76.8	137.6

The increasing flexibility of the D107G mutant further highlighted the importance of Thr_123_. The trajectories of the T123H mutant revealed less deflection of the THN motif and the closer proximity of Asp_107_ and Asn_125_ (Figure 5B), as well as D107K mutant showed a less deflection (Figure S12B, Supporting Information), which suggested that the absence of flexibility of block F is counterproductive to the deflection of the THN motif. A few prevalent interactions within the THN motif were detected in the D107K and T123H mutants (**Table** **3**), especially that between His_124_ and Asn_125_ (Figure 5C). The Thr_123_‐mediated deflection of the THN motif not only increased the distance between Asp_107_ and Asn_125_ but also separated the C‐extein and active site. Unlike that in Npu DnaE, no interactions were detected between the active site residue and the C‐extein in gp41‐1 (Figure S14A,B, Supporting Information), which suggested that the interaction between the C‐extein and His residue might restrict traceless cleavage.^[^ ^12^ ^]^ Furthermore, the hydrogen bond between the Thr_123_ side chain and main chain of His_124_ was key to stabilizing the THN motif to ensure the interaction between His_124_ and Asn_125_, since a disrupted THN motif was identified in inactive mutants (Figure 5B; Figure S14D, Supporting Information). We proposed that this interaction was prevalent in inteins that possess THN or SHN motifs; however, the interaction between Thr_123_ and His_124_ was observed only in class 3 inteins. The interaction between Thr_123_ and His_124_ stabilized the THN motif and thus separated Asp_107_ from Asn_125_, promoting the interaction between His_124_ and Asn_125_. Notably, the His_63_ and His_124_ interaction was observed in the T123A mutant, suggesting that His_63_ forms a new hydrogen bond with His_124_ to stabilize the structure of the THN motif if Thr_123_ is absent (Figure S14C, Supporting Information). However, the loss of this hydroxyl group results in a 50% reduction in cleavage activity. These results further highlighted that Thr_123_ is important not only for traceless cleavage but also for its rapid cleavage activity.

**Table 3:** Interactions between active site residues.

Intein	Thr_123_ and His_124_	His_124_ and Asn_125_	His_63_ and His_124_	Asp_107_ and Asn_125_ [Å]
gp41–1	+	+	–	10.7
D107Ggp41–1	+	+	–	NA
D107Kgp41‐1	+	–	–	NA
T123Agp41–1	–	+	+	10.7
T123Hgp41–1	–	–	–	7.7

gp41‐1‐GST/MBP presented similar MD results. Compared with gp41‐1, D107Ggp41‐1 presented consistent χ2 angles when fused to GFP, GST or MBP (Table 2), which suggested that the motion of the D107Ggp41‐1 active sites was similar even when it was fused to diverse proteins. In addition, we observed that the active sites of gp41‐1 would not be affect by it fusion proteins (Figure S15, Supporting Information). Our results further proved that the deflection of the THN motif in the engineered gp41‐1 allowed the C‐terminal cleavage process to not be affected by the extein residues.

## 3 Conclusion

In summary, the hydroxyl group of the unique residue Thr_123_ of gp41‐1 facilitates rapid, traceless C‐cleavage and significantly alleviates sequence constraints. The results suggest that the interplay between Thr_123_, His_124_ and Asn_125_ has direct implications for identifying inteins with high traceless cleavage activity. Moreover, increasing the flexibility of block F led to better C‐cleavage activity, as the D107G mutation further increased this activity. Furthermore, mutations at this site greatly affected the deflection of the THN motif and therefore inhibited the C‐cleavage process, indicating that the conformation of the THN motif was associated with traceless C‐cleavage activity. Therefore, the THN motif might be a target for the development of inteins with increased activity and promiscuity. These findings establish a structural and mechanistic framework for the rational engineering of inteins with enhanced C‐terminal traceless cleavage efficiency, offering broad applicability in protein biotechnology.

## Conflict of Interest

The authors declare no conflict of interest.

## Author Contributions

R.C.G., L.L.M., and H.Y.Y. conceived the project. R.C.G., Y.N.L., and R.Y.D. constructed plasmid and performed C‐cleavage assay in vitro. R.C.G., W.L., and L.L.M. analyzed the sequences and structures. R.C.G., T.T.Z., and T.W.F. analyzed the cleavage data and performed LC‐MS. R.C.G., and L.L.M. analyzed the MD stimulations. R.C.G. main‐authored the manuscript, Y.N.L., R.Y.D., T.T.Z., W.L, T.W.F., L.L.M., and H.Y.Y. co‐contributed to the manuscript.

## Supporting information



Supporting Information

Supporting Information

## Data Availability

The data that support the findings of this study are available from the corresponding author upon reasonable request.
