# Supplementary material for: A Unique THN Motif Is Critical for Enabling Efficient C‐Terminal Traceless Cleavage
Source: Adv Sci (Weinh). 2025 Apr 7;12(26):2501991. doi: 10.1002/advs.202501991 (PMC12245125; doi:10.1002/advs.202501991)
Supplement: Supplementary file 1 — Supporting Information [file ADVS-12-2501991-s001.docx]

Supporting Information

For

A Unique THN Motif Is Critical for Enabling Efficient C-Terminal Traceless Cleavage

**Ruocheng Gu^[a] [b]^, Yunuo Lin^[a] [b]^, Rouyu Di^[a] [b]^, Tongtong Zhou****^[a] [b]^, Tingwen Fan^[a] [b]^, Wei Li^[a] [c]^, Lili Miao^[a] [c]^*, Huaiyi Yang^[a] [b] [c]^***

**[a] Department of Microbial Physiological & Metabolic Engineering, State Key Laboratory of Microbial Diversity and Innovative Utilization, Institute of Microbiology, Chinese Academy of Sciences, Beijing 100101, China**

**miaoll@im.ac.cn,** **yanghy@im.ac.cn.**

**[b] University of Chinese Academy of Sciences, Beijing, 100049, China**

**[c] Beijing Key Laboratory of Genetic Element Biosourcing & Intelligent Design for Biomanufacturing, Beijing 100101, China**

**Experimental Section:**

**Materials**

Isopropyl-β-D-thiogalactopyranoside (IPTG) and all of the buffer salts were purchased from Thermo Scientific. Kanamycin sulfate (Kan) and DTT were purchased from Sigma-Aldrich. Coomassie brilliant blue was obtained from Beyotime (China). High-fidelity PCR enzymes and homologous recombination enzymes were purchased from Yeason (China). The gel extraction kit and plasmid extraction kit were procured from OMEGA. Complete protease inhibitor tablets were purchased from MedChemExpress (China). A Ni-NTA pretreated column was purchased from GE Healthcare. *Escherichia coli* DH5α (TSINGKE (Beijing, China)) was used for recombinant protein plasmid cloning, and *E. coli* BL21 (DE3) (TSINGKE (Beijing, China)) was used for recombinant protein expression. All the plasmids were sequenced by Sangon (China).

**Equipment**

Proteins were purified with an ÄKTA FPLC system (GE Healthcare). The molecular weights were calculated by SYNAPT XS (Waters). The gel bands were quantified with ImageJ. PyMOL was used to visualize the structures of the proteins.

**Cloning the gp41-1-GFP/GST/MBP expression plasmids**

gp41-1 (C1A) was amplified from the cloning vector, whereas GFP, GST and MBP were amplified from the expression vectors preserved in the laboratory. The gp41-1 proteins were subsequently fused to GFP, GST, and MBP by overlap PCR. The fusion nucleotides were subsequently cloned and inserted into the pET28 (a+) vector with a His_6_ tag (CACCACCACCACCACCAC) at the 5’ end of the multiple cloning site by overlap extension PCR. The plasmids pET28-gp41-1-GFP, pET28-gp41-1-GST, and pET28-gp41-1-MBP encoded the following protein sequences:

MGSSHHHHHH**ALDLKTQVQTPQGMKEISNIQVGDLVLSNTGYNEVLNVFPKSKKKSYKITLEDGKEIICSEEHLFPTQTGEMNISGGLKEGMCLYVKEMMLKKILKIEELDERELIDIEVSGNHLFYANDILTHN**MRKGEELFTGVVPILVELDGDVNGHKFSVRGEGEGDATNGKLTLKFICTTGKLPVPWPTLVTTLTYGVQCFARYPDHMKQHDFFKSAMPEGYVQERTISFKDDGTYKTRAEVKFEGDTLVNRIELKGIDFKEDGNILGHKLEYNFNSHNVYITADKQKNGIKANFKIRHNVEDGSVQLADHYQQNTPIGDGPVLLPDNHYLSTQSVLSKDPNEKRDHMVLLEFVTAAGITHGMDELYK

His_6_-gp41-1-GST

MGSSHHHHHH**ALDLKTQVQTPQGMKEISNIQVGDLVLSNTGYNEVLNVFPKSKKKSYKITLEDGKEIICSEEHLFPTQTGEMNISGGLKEGMCLYVKEMMLKKILKIEELDERELIDIEVSGNHLFYANDILTHN**MSPILGYWKIKGLVQPTRLLLEYLEEKYEEHLYERDEGDKWRNKKFELGLEFPNLPYYIDGDVKLTQSMAIIRYIADKHNMLGGCPKERAEISMLEGAVLDIRYGVSRIAYSKDFETLKVDFLSKLPEMLKMFEDRLCHKTYLNGDHVTHPDFMLYDALDVVLYMDPMCLDAFPKLVCFKKRIEAIPQIDKYLKSSKYIAWPLQGWQATFGGGDHPPK

His_6_-gp41-1-MBP

MGSSHHHHHH**ALDLKTQVQTPQGMKEISNIQVGDLVLSNTGYNEVLNVFPKSKKKSYKITLEDGKEIICSEEHLFPTQTGEMNISGGLKEGMCLYVKEMMLKKILKIEELDERELIDIEVSGNHLFYANDILTHN**MKIEEGKLVIWINGDKGYNSLAEVGKKFEKDTGIKVTVEHPDKLEEKFPQVAATGDGPDIIFWAHDRFGGYAQSGLLAEITPDKAFQDKLYPFTWDAVRYNGKLIAYPIAVEALSLIYNKDLLPNPPKTWEEIPALDKELKAKGKSALMFNLQEPYFTWPLIAADGGYAFKYENGKYDIKDVGVDNAGAKAGLTFLVDLIKNKHMNADTDYSIAEAAFNKGETAMTINGPWAWSNIDTSKVNYGVTVLPTFKGQPSKPFVGVLSAGINAASPNKELAKEFLENYLLTDEGLEAVNKDKPLGAVALKSYEEELVKDPRIAATMENAQKGEIMPNIPQMSAFWYAVRTAVINAASGRQTVDEALKDAQT

C1A mutations, highlighted in red, were introduced to block N-terminal cleavage activity. The sequence of gp41-1 is highlighted in bold.

The single point D107G, D107K, D107C, T123A, T123H, T123S, and H63A mutations were introduced by overlap PCR. The protein sequences used were as follows:

His_6_-gp41-1 (Asp_107_ mutation)

MGSSHHHHHH**ALDLKTQVQTPQGMKEISNIQVGDLVLSNTGYNEVLNVFPKSKKKSYKITLEDGKEIICSEEHLFPTQTGEMNISGGLKEGMCLYVKEMMLKKILKIEELDERELIG/K/CIEVSGNHLFYANDILTHN**

His_6_-gp41-1 (His_63_ mutation)

MGSSHHHHHH**ALDLKTQVQTPQGMKEISNIQVGDLVLSNTGYNEVLNVFPKSKKKSYKITLEDGKEIICSEEALFPTQTGEMNISGGLKEGMCLYVKEMMLKKILKIEELDERELIDIEVSGNHLFYANDILTHN**

His_6_-gp41-1 (Thr_123_ mutation)

MGSSHHHHHH**ALDLKTQVQTPQGMKEISNIQVGDLVLSNTGYNEVLNVFPKSKKKSYKITLEDGKEIICSEEHLFPTQTGEMNISGGLKEGMCLYVKEMMLKKILKIEELDERELIDIEVSGNHLFYANDILS/A/HHN**

Mutations to residues His_63_, Asp_107_, and Thr_123_ are highlighted in red and underlined. All the plasmids were cloned and inserted into *E. coli* DH5α and extracted with a plasmid extraction kit following the manufacturer’s recommended protocol.

**Expression and purification the gp41-1 constructs**

The expression plasmids were subsequently transformed into competent *E. coli* BL21 (DE3). A single colony was grown in LB media supplemented with Kan (50 μg/mL) at 37 °C until the OD600 reached 0.6~0.8. Then, 0.2 mM IPTG was added for 12 h of incubation at 25 °C to induce protein expression. The cells were collected by centrifugation at 10,000 rpm for 10 min, and the cell pellets were resuspended in 10 mL of Buffer A (100 mM NaCl, 10 mM Tris-HCl, pH 8.0). The cells were lysed via ultrasonication (60% amplitude, 3 seconds pulses with 5 seconds off on ice for 15 min). The soluble fractions were collected after 30 min of centrifugation at 12,000 rpm. The fusion proteins were subsequently purified with a 5 mL His Trap^TM^ HP column (GE, Boston, USA) as follows. 1: The column was washed and equilibrated for 5 column volumes (CVs); 2: the fusion proteins were eluted at a flow rate of 1 mL/min; 3: the column was washed with Buffer A containing 20 mM imidazole for 5 CVs; and 4: the target proteins were eluted with Buffer A containing 200 mM imidazole. The buffer in the fractions was exchanged for buffer composed of 25 mM Tris-HCl or 50 mM sodium acetate for subsequent cleavage experiments.

**Analysis of the C-terminal cleavage assay products by sodium dodecyl sulfate-polyacrylamide gel electrophoresis (SDS-PAGE) and liquid chromatography-mass spectrometry (LC-MS) and kinetic analysis**

The purified proteins were cleaved in the presence or absence of 20 mM DTT at 37 °C. Then, samples were collected at different time points for SDS-PAGE and LC-MS analyses. For SDS-PAGE, the samples were mixed with loading buffer and boiled at 100 °C for 10 min and then subjected to 12% SDS-PAGE. The gels were stained with Coomassie brilliant blue R250, the grey values were measured via ImageJ, and the data were analysed with Prism 9. The remaining cleavage samples were diluted to 100 ng/μL in 50 mM NH_4_HCO_3_ for ultra-performance liquid chromatography (UPLC) analysis. One hundred nanograms of sample was injected onto an ACQUITY UPLC I-Class PLUS system equipped with a BEH C4 column (130 Å, 1.7 μm, 2.1 × 50 mm; Waters, USA). Chromatographic separation was achieved using a linear gradient from 3% B to 97% B over 4.60 min at a flow rate of 200 μL/min and a column temperature of 80 °C. The mobile phases were 0.1% formic acid in water (A) and 0.1% formic acid in acetonitrile (B).

The MS experiments were performed using a SYNAPT XS mass spectrometer with a standard electrospray ionization (ESI) source (Waters, USA). The MS parameters were as follows: positive ESI mode; capillary voltage, 3.0 kV; cone voltage, 40 V; source heating temperature, 120 °C; desolvation temperature, 600 °C; cone gas flow, 100 L/hr; desolvation gas flow, 600 L/hr; mass/charge (m/z) range, 400-5000; and scan duration, 0.5 seconds. Lock mass data were collected during each acquisition, and the correction was applied during postacquisition data processing using MassLynx Software v4.1 (Waters, USA). The mass spectra were deconvoluted using MaxEnt1 in UNIFI software (Waters, USA). The rate constant was calculated by fitting the data to the equation P=P0(1-e^-kt^), where P is the percent cleavage product formation at time t, P0 is the maximum percent cleavage product obtained (yield), and *k* is the observed rate.

**MD simulations**

Molecular dynamics (MD) simulations were performed using GROMACS. The protonation states of ionizable amino acids were predicted using the H++ server at pH 6.0 to ensure an accurate representation of the protein's charge distribution under physiological conditions. The initial structure was prepared by converting the input PDB file to GROMACS format, employing the TIP3P water model and the AMBER99SB force field. A cubic simulation box was defined with a minimum distance of 1.0 nm between the protein and the box edges. The system was solvated with water molecules, and ions (sodium, Na⁺, and chloride, Cl⁻) were added to neutralize the system. Energy minimization was performed using the steepest descent algorithm to relax the system. Subsequently, the system was equilibrated under the NVT (constant number of particles, volume, and temperature) and NPT (constant number of particles, pressure, and temperature) ensembles. Finally, a production MD simulation was conducted for 100 ns with a time step of 2 fs, periodic boundary conditions in all dimensions, and Particle Mesh Ewald (PME) electrostatics for long-range interactions. GPU acceleration was utilized to enhance computational efficiency.

**Statistical Analysis**

All data were repeated for at least three times, especially for cleavage data. Data were analysed by GraphPad Prism 9.0 software and presented as means ± SD. One way analysis of variance (ANOVA) was as necessary, with P < 0.05 being statistically significant.

Figure S1

To analyse the traceless cleavage ability of gp41-1, we constructed Npu DnaE-GFP and Npu DnaE-CFN-GFP plasmids to prove that inteins do not commonly perform traceless cleavage. Npu DnaE could perform cleavage only when its native extein residues CFN were present; thus, performing traceless cleavage with Npu DnaE was difficult.


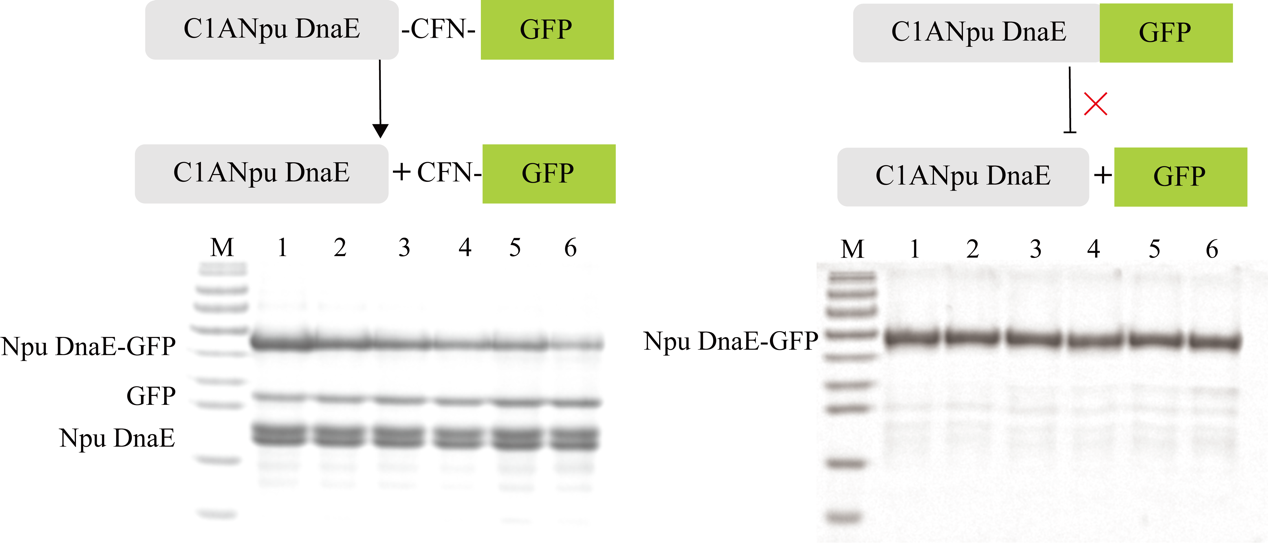


SDS-PAGE analysis of the cleavage products of Npu DnaE-CFN-GFP and Npu DnaE-GFP

Lanes 1-6 are samples taken after 0 h, 3 h, 6 h, 9 h, 12 h and 24 h of continuous cleavage. Npu DnaE could perform C-cleavage only when the CFN sequence was present.

Figure S2

It has been reported that the C1A intein mutant might retain some N-terminal cleavage activity^[1]^. We hence constructed a Trx-gp41-1-GFP expression vector to investigate whether the trans-splicing reaction was completely abolished with C1Agp41-1. SDS-PAGE revealed that no Trx-GFP side product (~ 38 kDa) was produced during cleavage. Thus, N-terminal activity was completely abolished in C1Agp41-1, laying a foundation for its application in protein purification and tag removal.


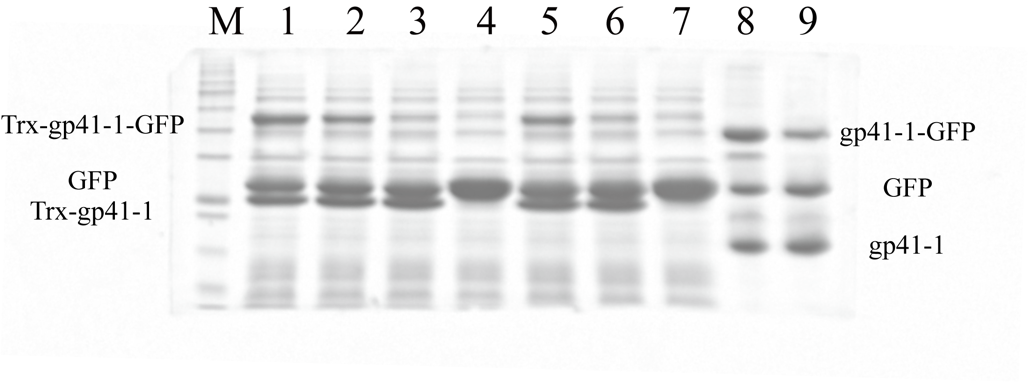


SDS-PAGE analysis of Trx-gp41-1-GFP cleavage.

M: Marker; 1-4: Trx-gp41-1-GFP cleavage for 0 h, 6 h, 12 h, and 24 h; 5-7: duplicate samples taken at 6 h, 12 h, and 24 h; and 8-9: His-gp41-1-GFP cleavage for 0 h and

12 h. No splicing products were detected.

Figure S3

The raw data of LC-MS was shown in Fig. S3. Three major proteins were detected in LC-MS which was consistent with SDS-PAGE. The major protein was gp41-1-GFP (~ 44.3 kDa), followed by gp41-1 and the fusion tag at N-terminus (17.5 kDa), only a few GFP (26.7 kDa) were detected in 0 h. The cleavage products (gp41-1 and GFP) accumulated with the cleavage time, while the intensity of fusion proteins (gp41-1-GFP) decreased.


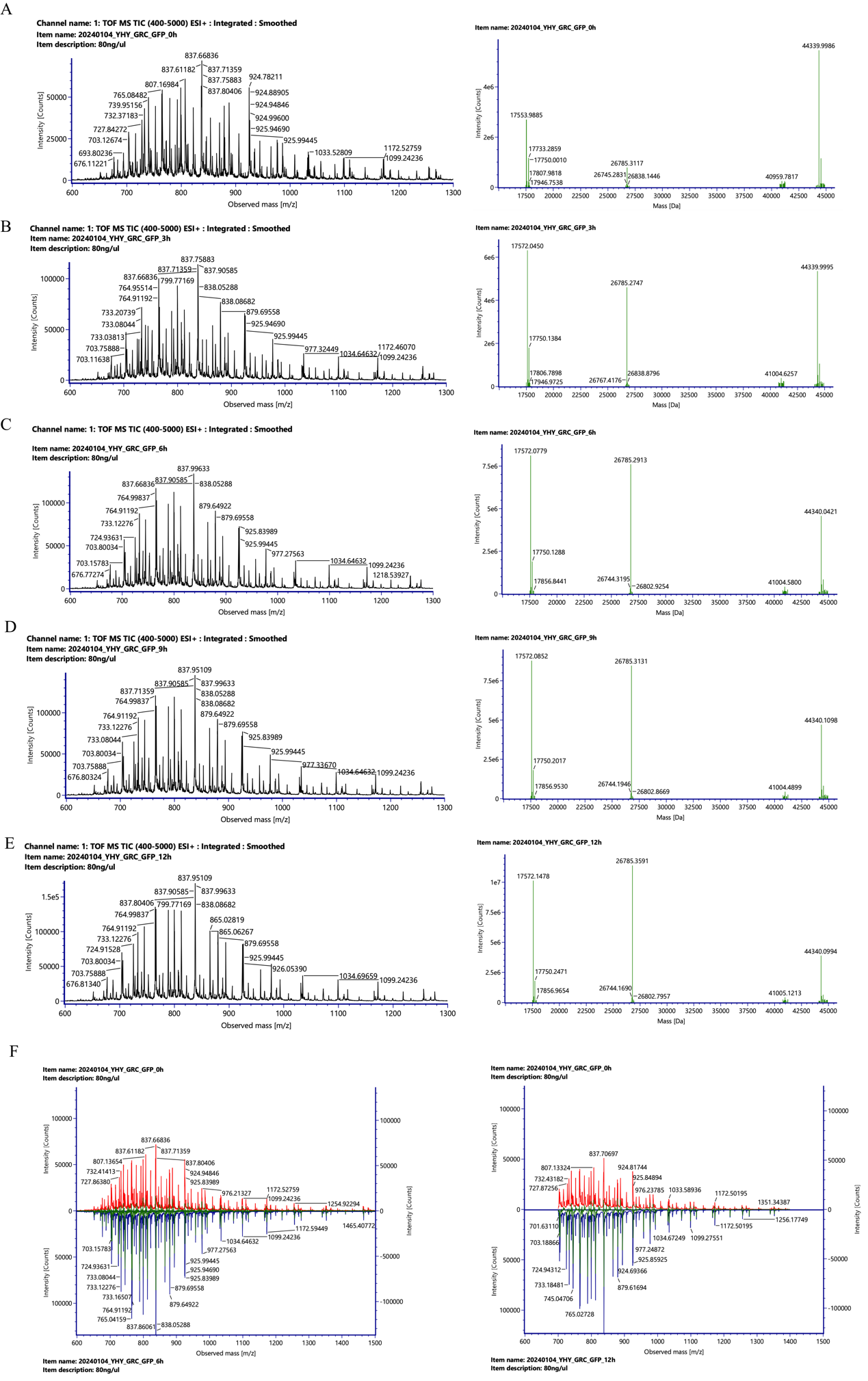


LC-MS analysis of gp41-1-GFP cleavage process

A-E) The analysis of cleavage products in 0 h, 3 h, 6 h, 9 h and 12 h, Three major peaks represented cleavage products including gp41-1 (~ 17.5 kDa), GFP (~ 26.7 kDa) and fusion proteins gp41-1-GFP (~ 44.3 kDa); F) The comparisons of m/z between 0 h and 6 h, and 0 h to 12 h respectively, an obvious increasing peaking was observed with cleavage time.

Figure S4

Unexpected cleavage is always concerned about, if intein was expressed as a continuous intein. We noticed unexpected cleavage in vivo, and the unexpected cleavage rate was consistent with the cleavage activity. Changing culture condition (lower temperature) could alleviate unexpected cleavage to 20% for gp41-1, 17% for T123Agp41-1, 33% for D107Ggp41-1 and 20% for T123Sgp41-1.


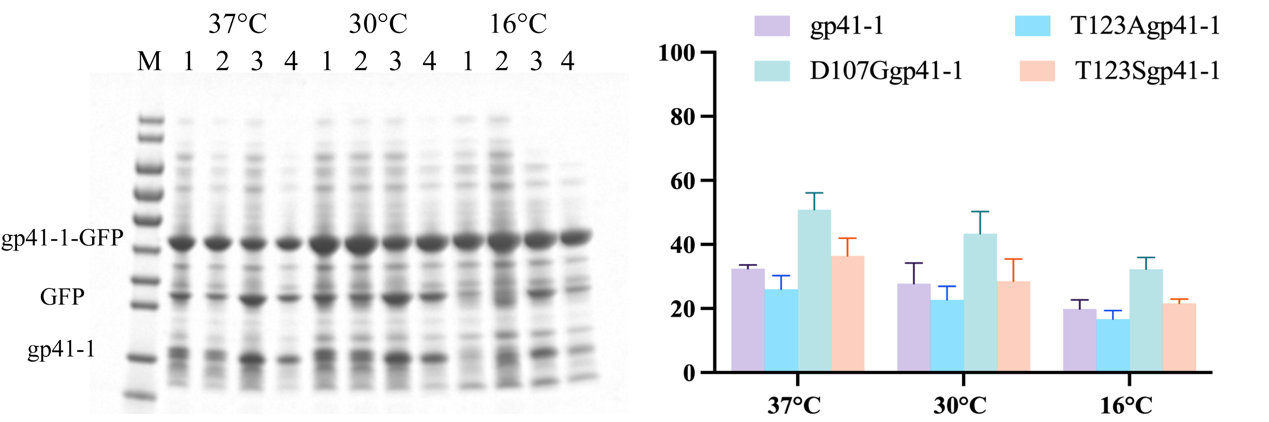


Unexpected cleavage of gp41-1 and its mutants under different culture condition

M: Marker; 1: gp41-1; 2: T123Agp41-1; 3: D107Ggp41-1; 4: T123Sgp41-1.

Figure S5

Compared with other classic inteins, gp41-1 presented unique abilities. We aligned the sequence of gp41-1 to those of other inteins, including Npu DnaE^[2]^, Ssp DnaE^[3]^, mini-RecA^[4]^ and the class 3 intein MchDnaB1^[5]^. Intein gp41-1 presented a unique sequence characteristic. Gp41-1 has some sequence features of class 3 inteins (with a difference in the TXXH motif, which is conserved as THN in class 3 inteins) but possesses a block F that is similar to those of class 1 inteins (with a conserved Asp residue). We further overlaid the structures of different inteins and found that the distance between Asp_118_ and Asn_137_ was 1.9 Å in the Npu DnaE structure and 3.2 Å in the DnaX structure, whereas the corresponding distance in the gp41-1 structure was greater than 7 Å, and a water molecule was within the hydrogen bonding distance between Asp_107_ and Asn_125_. This result indicated that the long distance between Asp_107_ and Asn_125_ caused by the THN motif was responsible for the high cleavage activity of gp41-1.


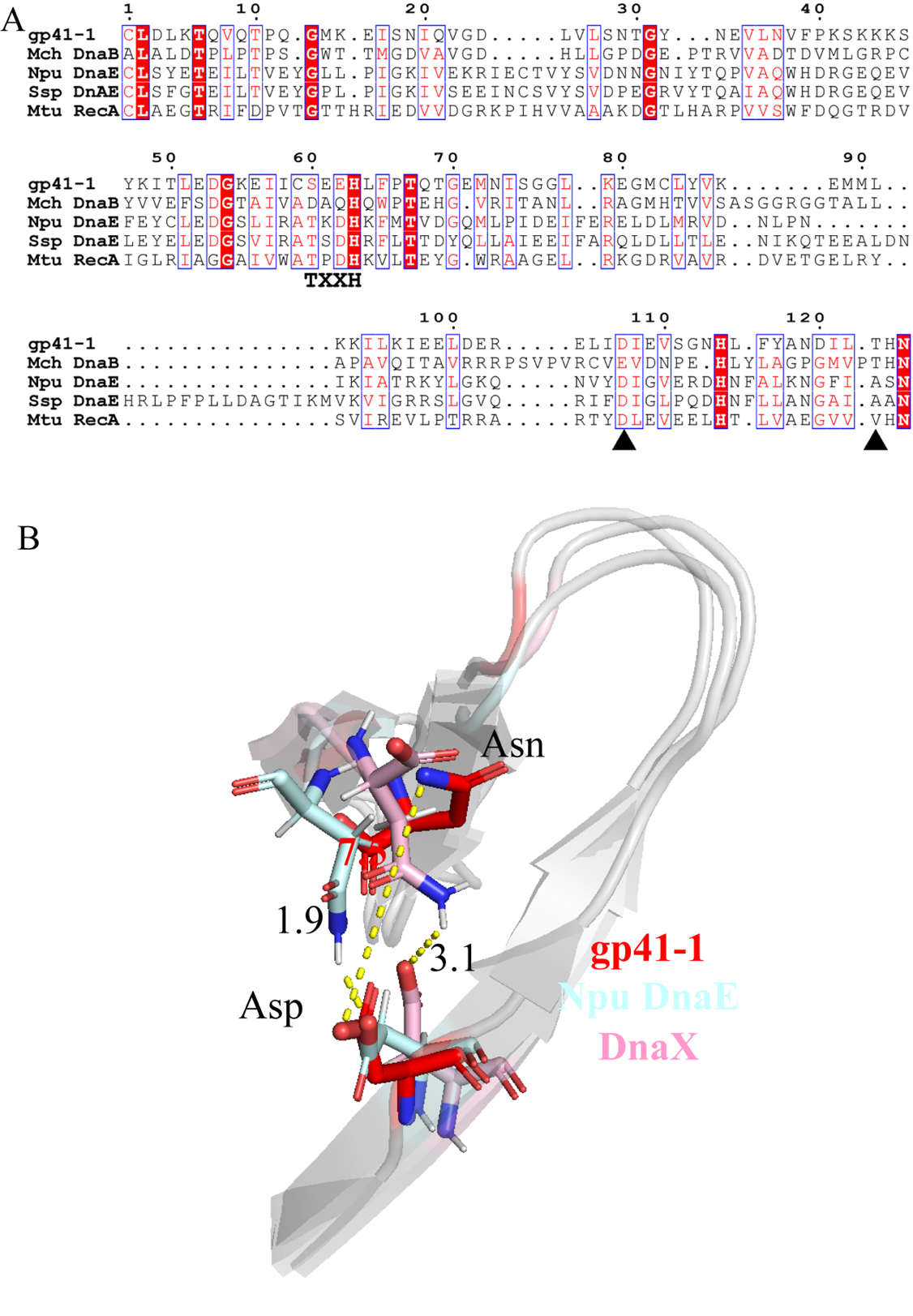


Sequence and structural alignments of gp41-1 with other inteins.

A) Sequence alignment of gp41-1 with other inteins. Gp41-1 has a different block B sequence, SXXH, and the presence of the unique residue Thr_123_. B) Hydrogen bonds between the Asp and Asn residues of gp41-1, Npu DnaE and DnaX; only Asp_107_ and Asn_125_ of gp41-1 cannot interact directly.

Figure S6

Mutation of Thr_123_ to His or Asp strongly inhibited C-cleavage activity, with cleavage rates of < 25% after 24 h. These data suggested that the steric hindrance of His and Asp at this position might be responsible for the reduced cleavage activity.


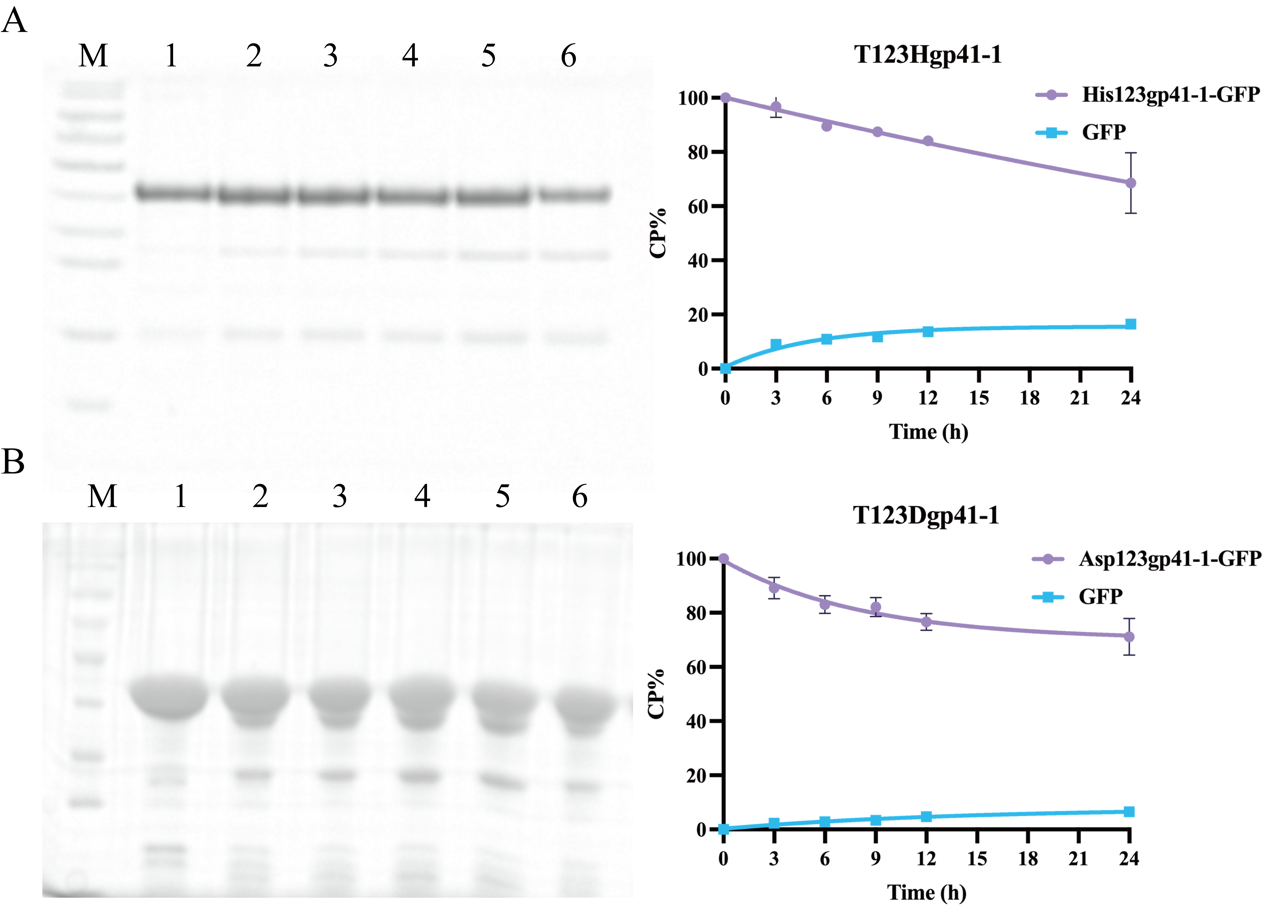


C-cleavage activities of the T123H and T123D mutants.

A) SDS-PAGE analysis of the T123H mutant C-cleavage products. B) SDS-PAGE analysis of the T123D mutant C-cleavage product.

Figure S7

The T123A mutant was fused to GFP with different +1 residues. The cleavage data revealed that the cleavage activity decreased in the presence of different +1 residues. These results further indicated that the hydroxyl group of Thr_123_ contributed to cleavage.


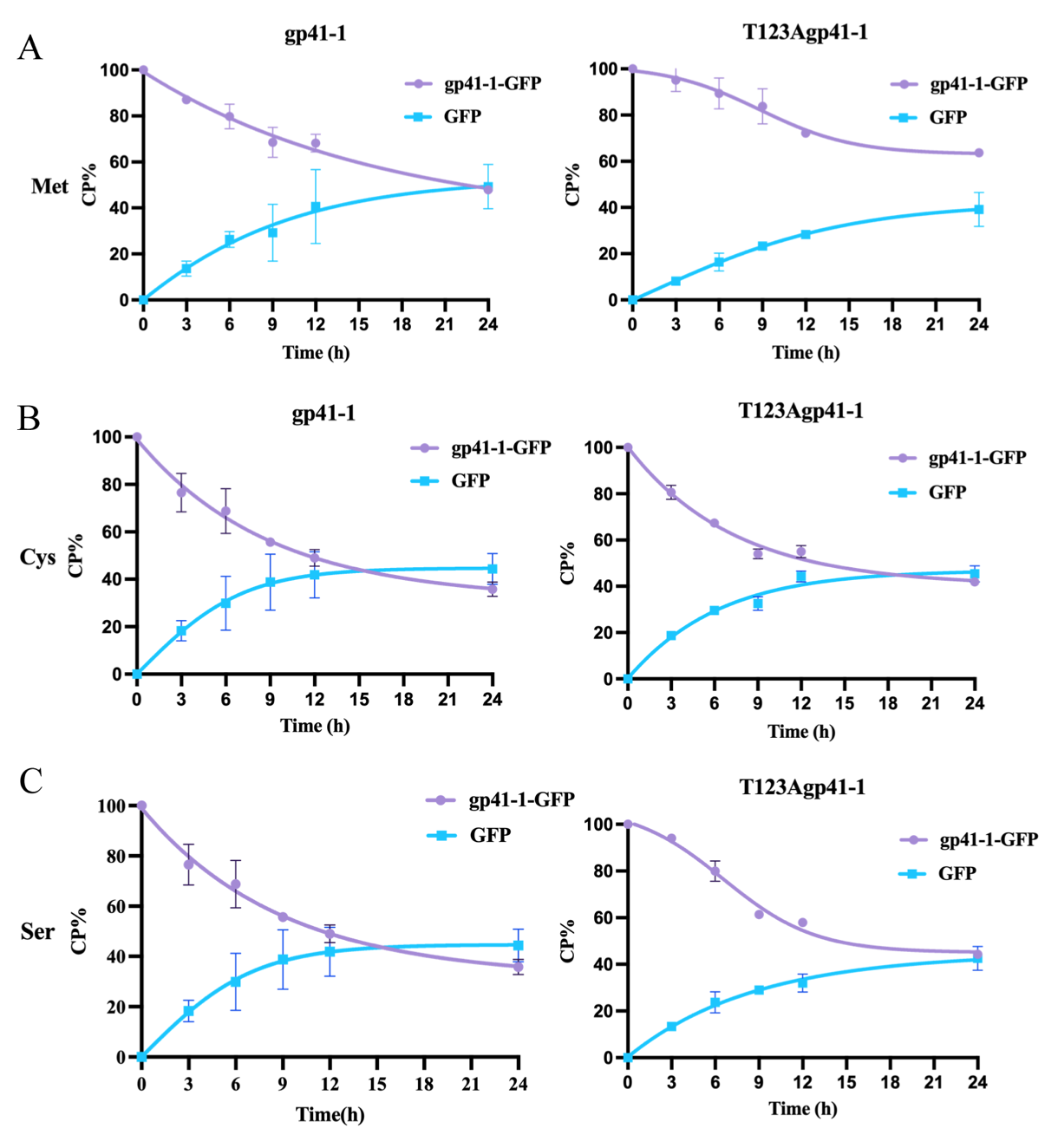


A) Cleavage products of gp41-1 and the T123A mutant with Met as the +1 residues. B) Cleavage products of gp41-1 and the T123A mutant with Cys as the +1 residues. C) Cleavage products of gp41-1 and the T123A mutant with Ser as the +1 residues. T123A mutant exhibited substantially decreased cleavage activity.

Fig. S8

We predicted and aligned the structures of gp41-1 (red) and other inteins containing the THN/SHN motif (grey). These inteins share similar structures that differ in the distance between the Asp in block F and the Asn at the C-terminus. These results indicated that Thr_123_ was responsible for increasing the distance between these two residues and thus increasing cleavage activity.


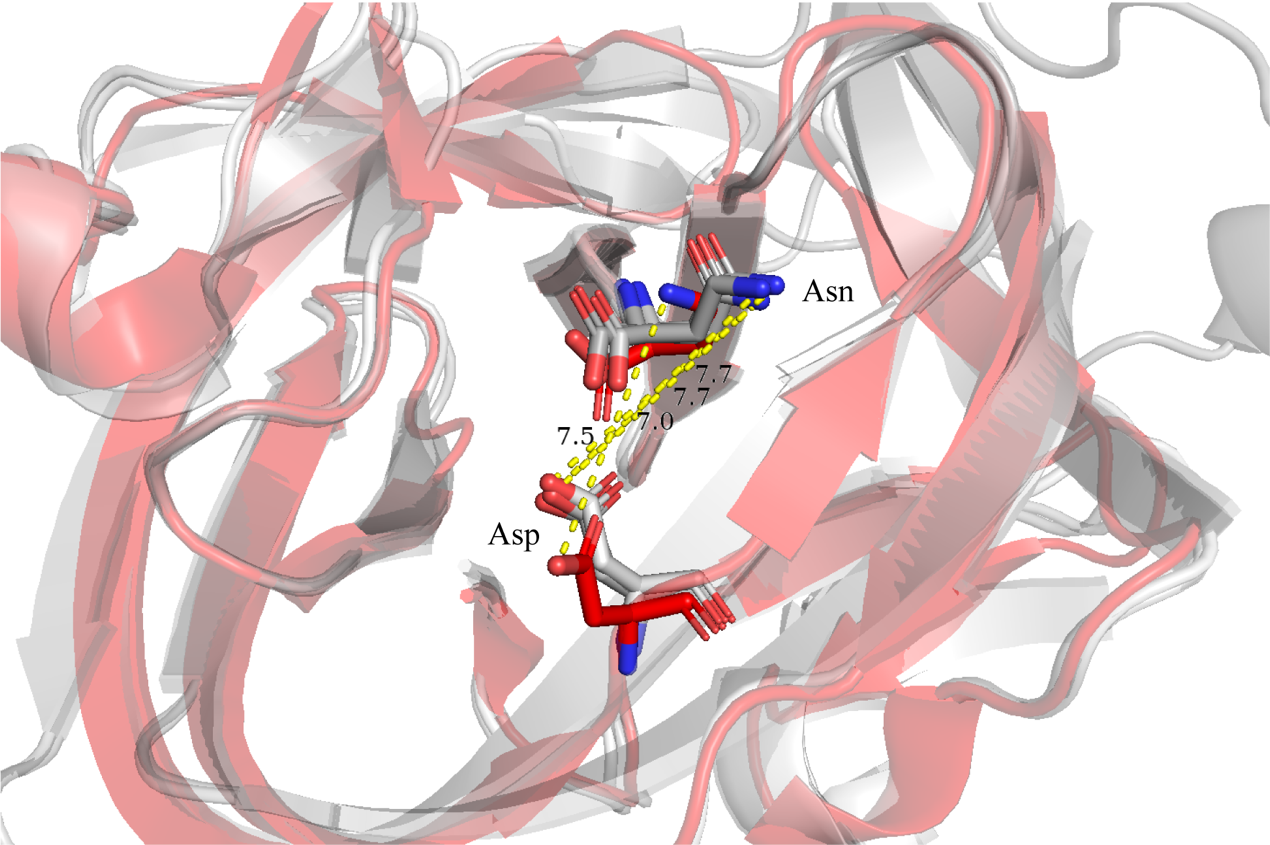


Distance between the Asp and Asn residues in inteins with THN/SHN motifs.

Gp41-1 and other inteins with THN/SHN motifs displayed similar distances between the Asp and Asn residues (approximately 7.5 Å).

Figure S9

We hypothesized that Thr_123_ provided its hydroxyl group to facilitate C-terminal cleavage. We then performed in vitro cleavage reactions without DTT with all the constructs. SDS-PAGE revealed that cleavage with gp41-1 and its mutants could occur without DTT. However, the T123A mutant presented significantly decreased cleavage activity without DTT, whereas the T123S mutant presented cleavage activity similar to that of gp41-1. These results further indicated that the hydroxyl group of Thr_123_ promoted cleavage activity.


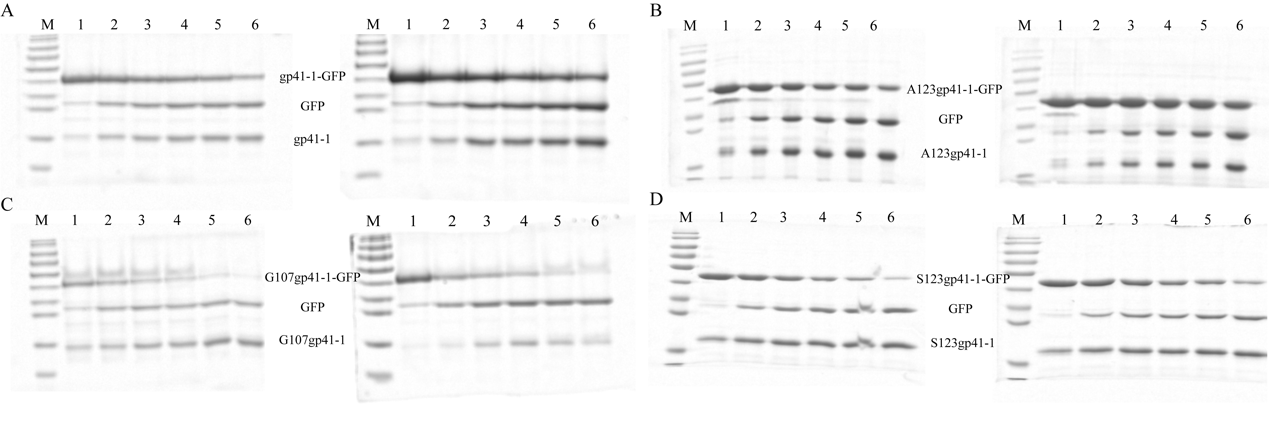


SDS-PAGE analysis of the cleavage products of gp41-1 and its mutants with and without DTT. Cleavage with DTT is shown on the left, whereas cleavage without DTT is shown on the right.

A) gp41-1. B) T123Agp41-1. C) D107Ggp41-1. D) T123Sgp41-1.

Figure S10

In the structure of gp41-1, a water molecule is within the hydrogen bonding distance between Asp_107_ and Asn_125_ or His_63_^[6]^. Our results revealed that mutation of Asp_107_ to Gly breaks this hydrogen bond and increases the cleavage activity. To determine which hydrogen bond is responsible for inhibiting the cleavage process, we made a single point mutation: H63A. Interestingly, the activity of the H63A mutant was similar to that of gp41-1. The His residue in block B is considered involved in the process of N-S/O acyl shift and transesterification^[7]^. We therefore hypothesized that His_63_ in gp41-1 was important for splicing.


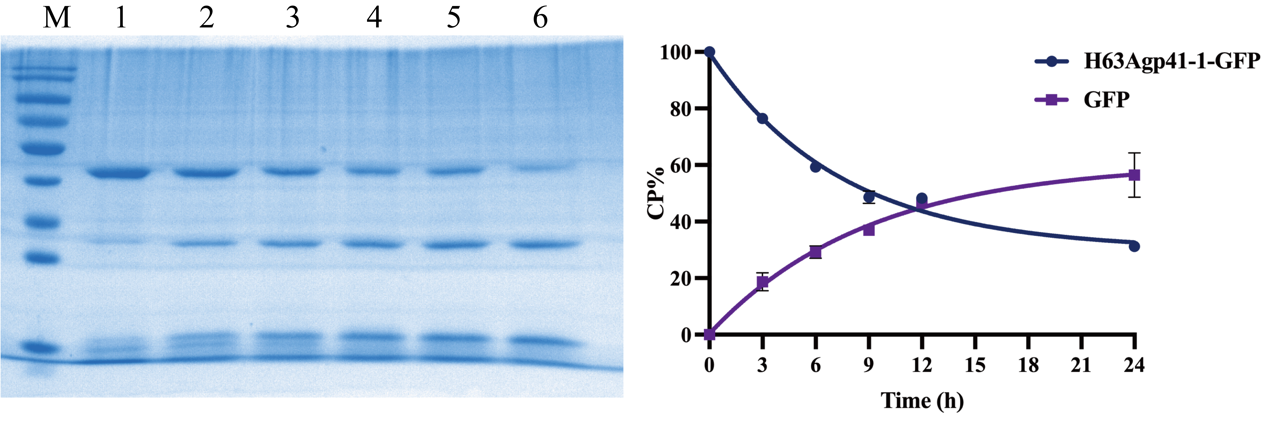


Cleavage activity of H63Agp41-1 in vitro.

SDS-PAGE analysis of the C-cleavage products of H63Agp41-1 in vitro at different time points (left). Lanes 1-6: 0 h-24 h. Rate of H63Agp41-1-GFP cleavage (right).

Figure S11

Traceless protein cleavage remains a challenge during intein application. We first used the GFP target protein to investigate the traceless cleavage activity of gp41-1. As shown in the image, gp41-1 could perform traceless cleavage, and the cleavage activity of G107gp41-1 was higher than that of gp41-1, reaching almost 90% after 3 h (Fig. 2A). To determine of gp41-1 could also perform traceless cleavage upon fusion to other proteins, we constructed the fusion proteins gp41-1-GST and gp41-1-MBP. SDS-PAGE analysis of the products showed results similar to those with gp41-1-GFP under the same conditions. Thus, gp41-1 was confirmed to perform traceless cleavage, with the D107G mutant showing increased activity. These results revealed the great potential of gp41-1 for protein purification and tag removal applications.


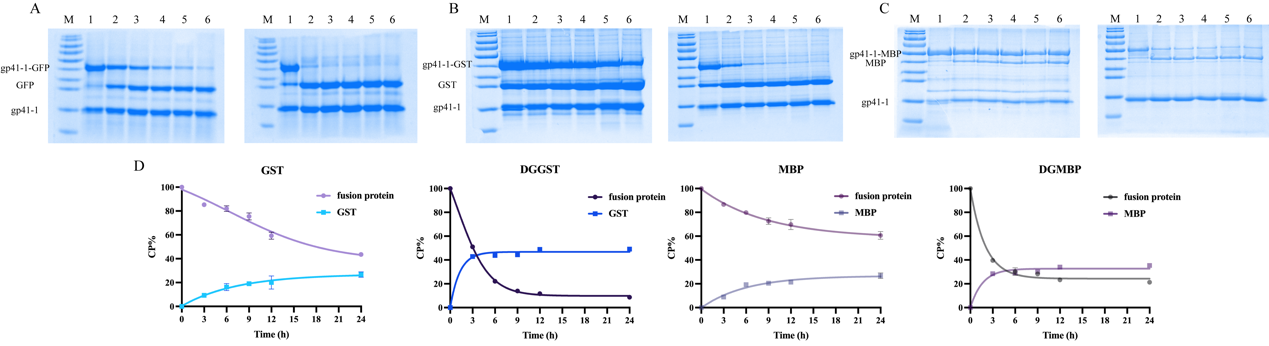


SDS-PAGE analysis of samples collected during cleavage with 40 mM DTT; the amount of protein loaded in each lane was the same. Lanes 1-6: 0 h, 3 h, 6 h, 9 h, 12 h and 24 h.

A) gp41-1-GFP cleavage. B) gp41-1-GST cleavage. C) gp41-1-MBP. D: Comparison of cleavage with gp41-1 and D107Ggp41-1.

Figure S12

The flexibility of block F and the cleavage activity increased with the D107G mutant. To further prove the impact of flexibility on cleavage activity, a compact residue was introduced into block F. The C-cleavage data revealed greatly reduced cleavage activity with the D107K mutant, which further proved that flexibility in block F was beneficial for the cleavage process. Moreover, MD simulations revealed that both the T123H and D107K mutants presented a disturbed motif. This result suggested that keeping a proper residue at the 107^th^ and 123^rd^ positions was important to maintain cleavage activity.


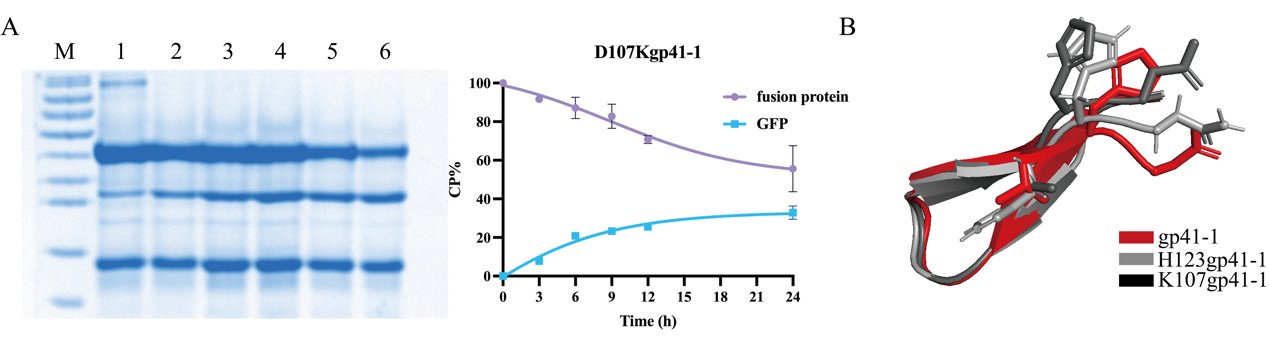


Cleavage assay with the D107K mutant and its structural alignment with gp41-1.

A) The D107K mutant showed inhibited cleavage activity, suggesting the importance of the flexibility of block F. B) Both the D107K and T123H mutants presented a disturbed motif.

Figure S13

Since gp41-1 and its mutants presented completely different trajectories, we analysed the structure of the THN motif. Notably, the flexibility of the THN motif increased in the D107G mutant with a disordered His_124_, whereas gp41-1 and the other mutants presented relatively compact THN motifs. This result suggested that a flexible THN motif was beneficial for the rapid interaction between His_124_ and Asn_125_.


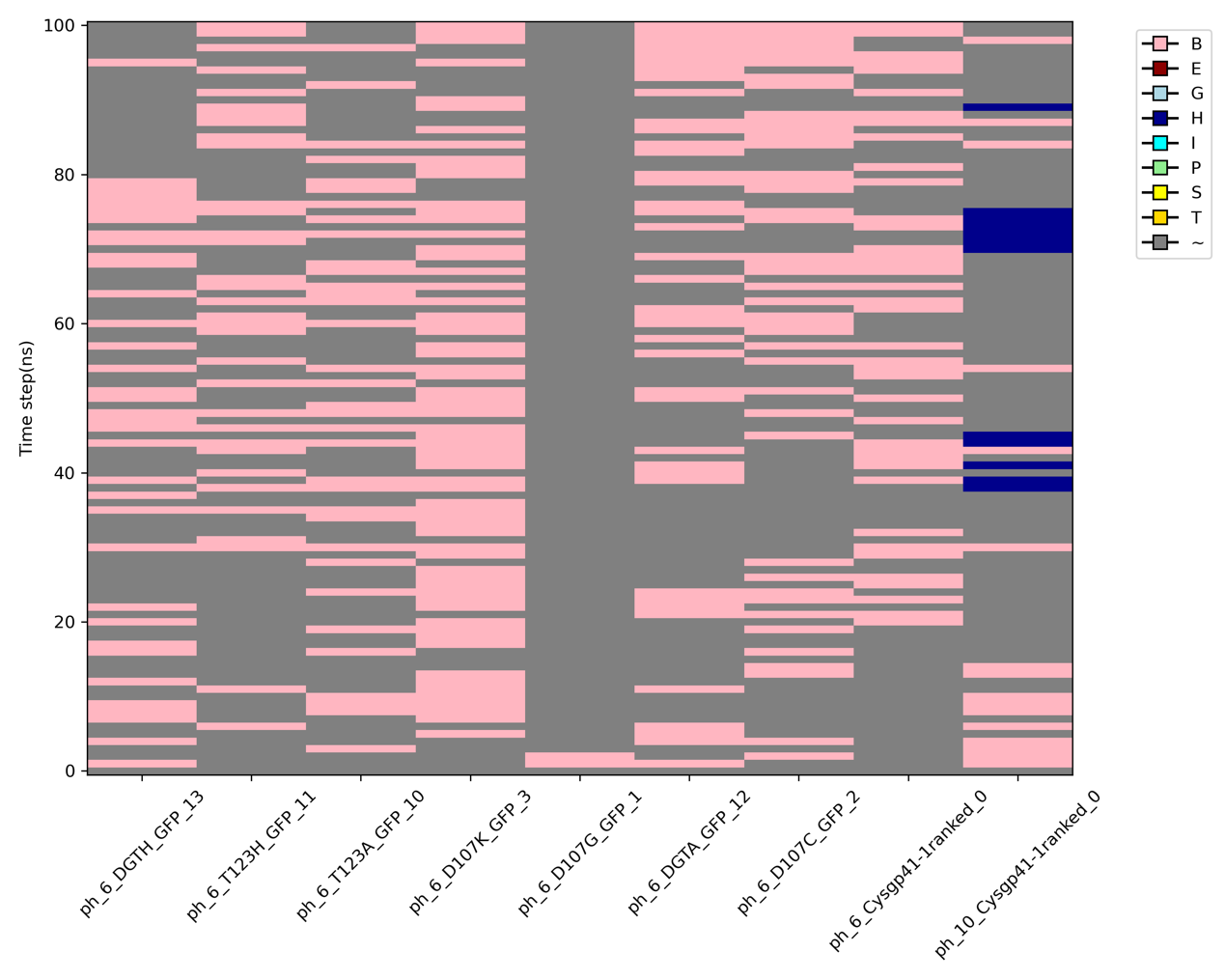


The flexibility of His_124_ in gp41-1 and its mutants.

The D107G mutant presented the most flexible THN motif.

Figure S14

The interactions between C-exteins and active sites of gp41-1 were detected. We found the deflection of THN motif avoid interference from C-extein, whereas HHN motif led to an interaction between C-extein and Asn_125_. Our results indicated that the deflection of THN motif was the main reason for traceless cleavage ability of gp41-1.


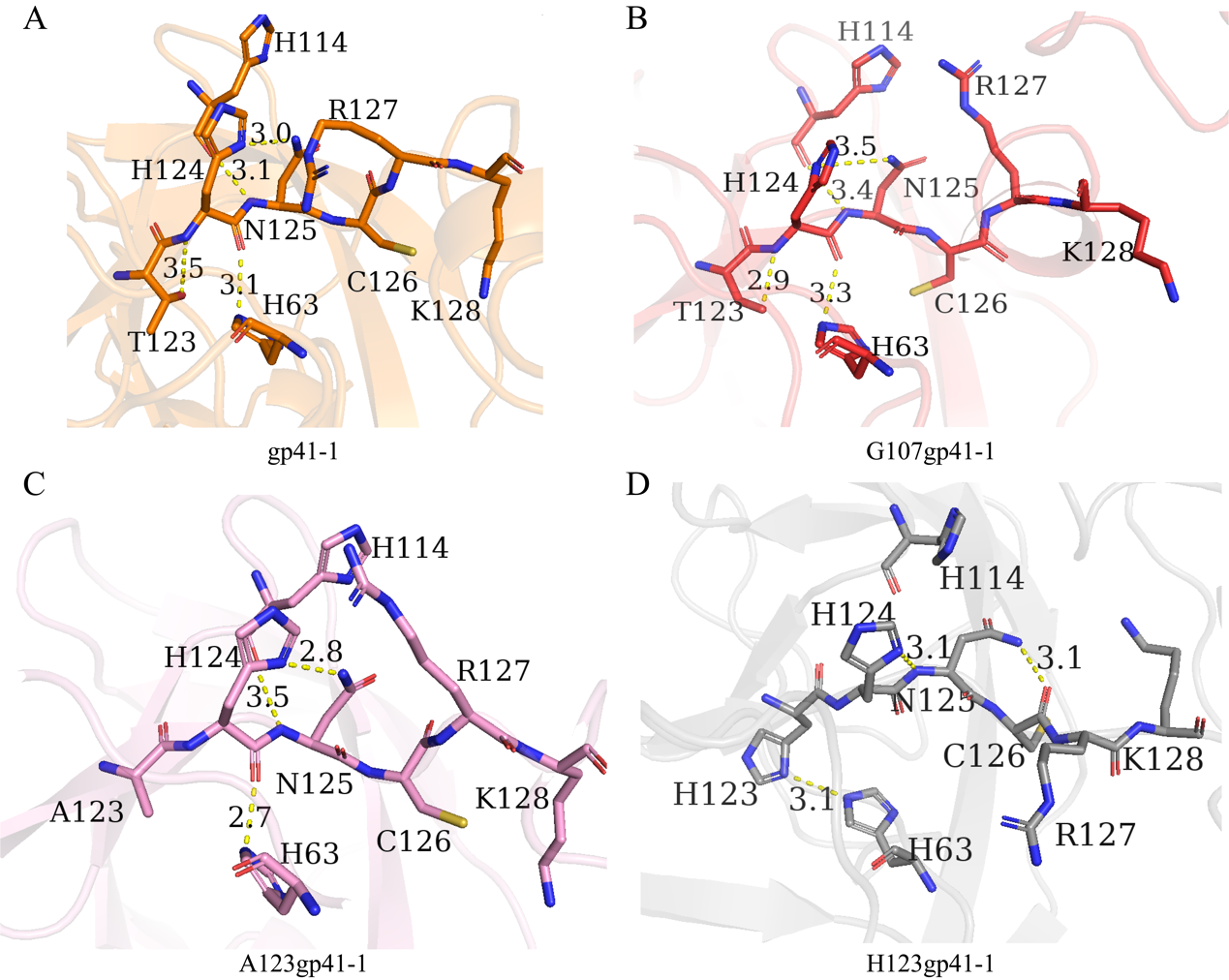


The interactions between C-exteins and active sites of gp41-1

A) Multiple interactions were detected in gp41-1, especially the interaction of Thr_123_ and His_124_, His_124_ and Asn_125_. B) A closer distance between Thr_123_ and His_124,_ longer distance of His_124_ and Asn_125_ was notice in G107gp41-1. C) A123gp41-1 maintained His_124_ and Asn_125_ interaction, while lacking the interaction of Thr_123_ and His_124_. D) No interaction within THN motif was detected, whereas an obvious interaction between Asn_125_ and C-extein was noticed. The deflection of THN motif avoided interference from C-extein,

Fig. S15

The effect of structures of target proteins was also concerned about. The MD stimulations showed that the target proteins stayed away from active sites of gp41-1, and therefore target proteins could hardly affect the cleavage activity of gp41-1. The diverse structures of GFP, GST and MBP further highlighted gp41-1-mediated expression system was universal for different proteins.


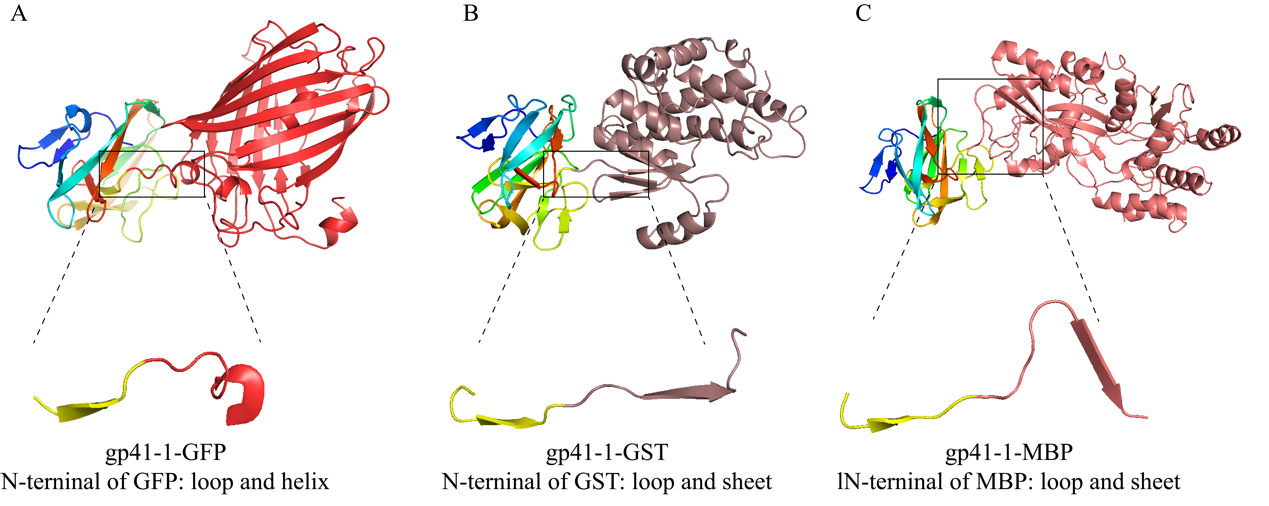


The structures of gp41-1 fusion proteins

A) The structure of gp41-1-GFP, the overall structure of GFP was β-sheet, while the junction structure was consisted of a loop and a helix. B) The structure of gp41-1-GST, the overall structure of GST was α-helix, while the junction structure was consisted of a loop and a sheet. C) The structure of gp41-1-MBP, the overall structure of GFP was consisted of α-helix and β-sheet, and the junction structure was consisted of a longer loop and a sheet.

Reference

[1] M. Ramirez, N. Valdes, D. Guan, Z. Chen, *Protein Eng Des Sel* **2013**, *26*, 215-223.

[2] H. Iwai, J. Z├╝ger S Fau - Jin, P.-H. Jin J Fau - Tam, P. H. Tam, *FEBS Lett* **2006**, Mar 20;580(587):1853-1858.

[3] P. Sun, S. Ye S Fau - Ferrandon, T. C. Ferrandon S Fau - Evans, M.-Q. Evans Tc Fau - Xu, Z. Xu Mq Fau - Rao, Z. Rao, *J Mol Biol* **2005**, *353(5)*, 1093-1105.

[4] P. Van Roey, B. Pereira, Z. Li, K. Hiraga, M. Belfort, V. Derbyshire, *Journal of Molecular Biology* **2007**, *367*, 162-173.

[5] D. S. Kelley, C. W. Lennon, Z. Li, M. R. Miller, N. K. Banavali, H. Li, M. Belfort, *Nature Communications* **2018**, *9*, 4363.

[6] H. M. Beyer, K. M. Mikula, M. Li, A. Wlodawer, H. Iwaï, *Febs j* **2020**, *287*, 1886-1898.

[7] K. Friedel, M. A. Popp, J. C. J. Matern, Emerich M. Gazdag, I. V. Thiel, G. Volkmann, W. Blankenfeldt, H. D. Mootz, *Chemical Science* **2019**, *10*, 239-251.
