# Supplementary material for: A Unique THN Motif Is Critical for Enabling Efficient C‐Terminal Traceless Cleavage
Source: Adv Sci (Weinh). 2025 Apr 7;12(26):2501991. doi: 10.1002/advs.202501991 (PMC12245125; doi:10.1002/advs.202501991)

Production Data

SDS-PAGE data


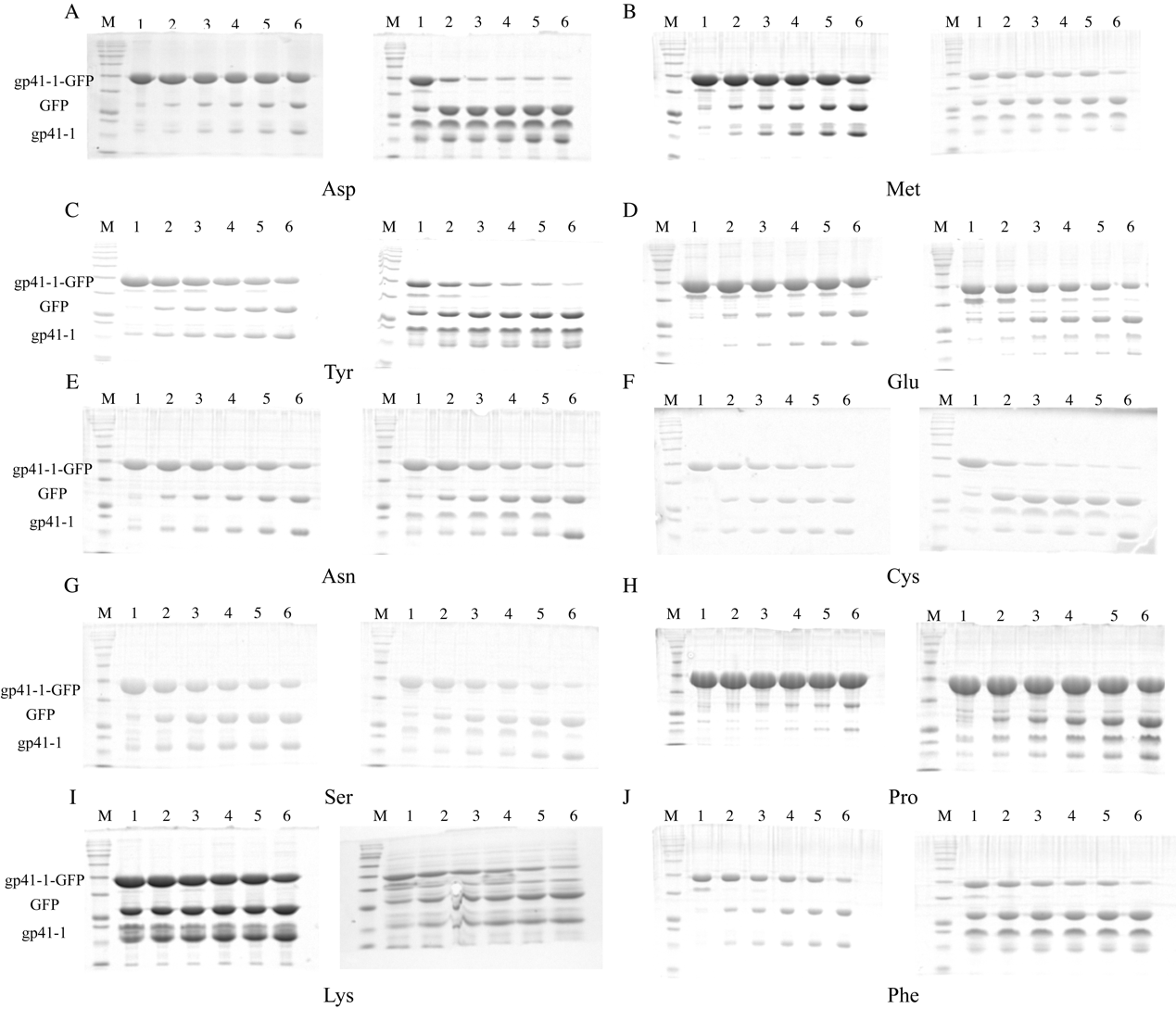


SDS-PAGE of each first amino acid (For Fig. 2D)

gp41-1 (left), D107Ggp41-1 (right), M: Marker; Lane 1-6: cleavage for 0 h, 3 h, 6 h,

9 h, 12 h and 24 h.


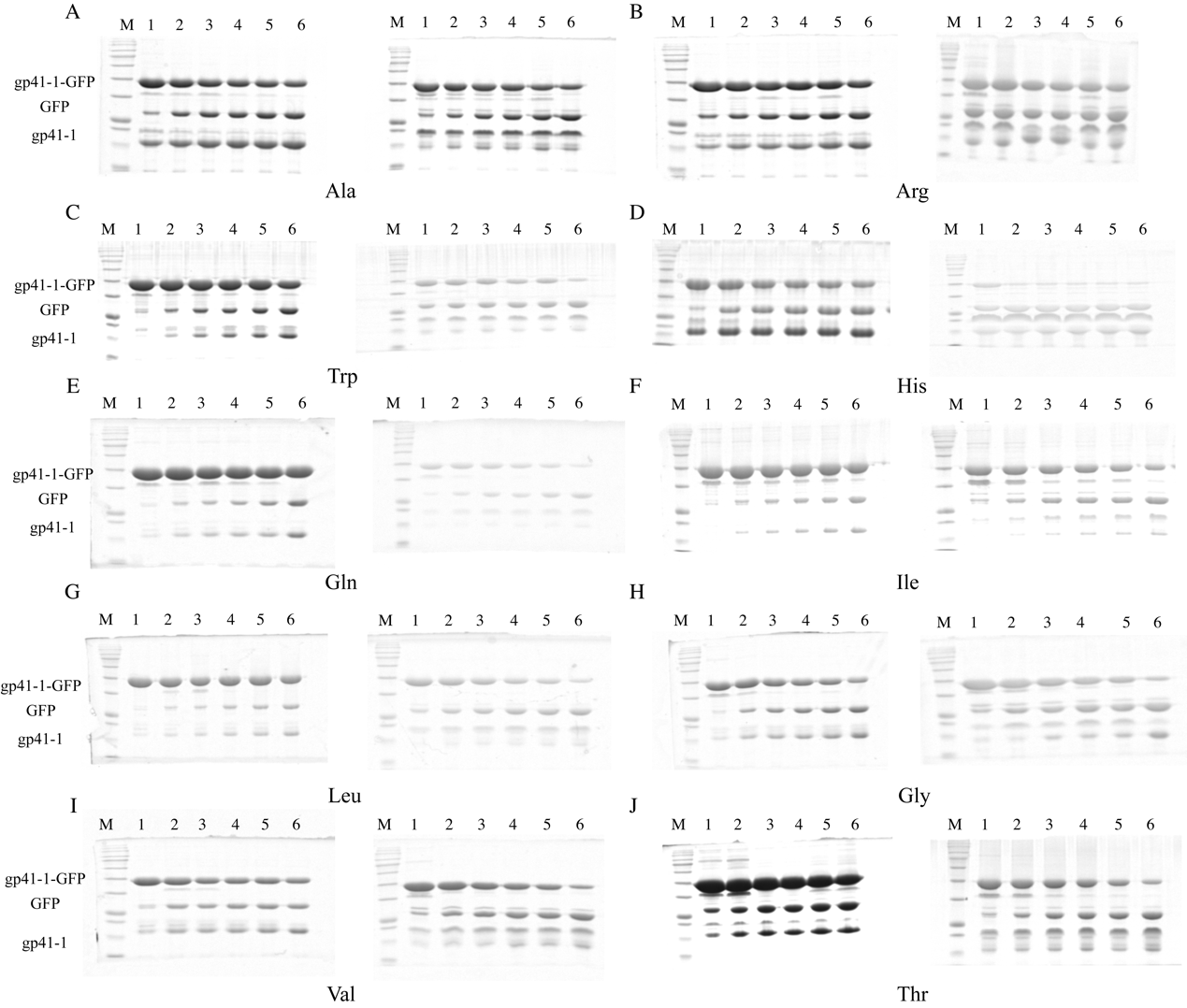


SDS-PAGE of each first amino acid (For Fig. 2D)

gp41-1 (left), D107Ggp41-1 (right), M: Marker; Lane 1-6: cleavage for 0 h, 3 h, 6 h,

9 h, 12 h and 24 h.

Cleavage efficiency of gp41-1 and D107Ggp41-1 after 24 h cleavage under pH 8

(For Fig. 2D)

| C+1 residue | nature gp41-1 | | | D107Ggp41-1 | | |
| --- | --- | --- | --- | --- | --- | --- |
| Ala | 66 | 60.36 | 62.5 | 82 | 79.4 | 80.33 |
| Arg | 36 | 39.58 | 37.31 | 52.31 | 58.75 | 55.7 |
| Asn | 59 | 47.92 | 53.78 | 73 | 72.44 | 75.28 |
| Asp | 24 | 17.4 | 20.35 | 81 | 77.28 | 80.44 |
| Cys | 67 | 65 | 70.3 | 88 | 86.43 | 89.66 |
| Gln | 33 | 35 | 33.6 | 53 | 56.54 | 55.98 |
| Glu | 7 | 17.39 | 15.6 | 84 | 72.44 | 81.75 |
| Gly | 56 | 53.33 | 55.69 | 69 | 65.67 | 66.3 |
| His | 60 | 60.29 | 63.58 | 79 | 71.68 | 72.45 |
| Ile | 32 | 23.54 | 29.5 | 61 | 57.28 | 58.56 |
| Leu | 48 | 39.62 | 44.1 | 69 | 66.99 | 67.87 |
| Lys | 36 | 44.46 | 38.5 | 50 | 49.95 | 53.6 |
| Met | 51.42 | 58.42 | 55.4 | 86.3 | 77.17 | 81 |
| Phe | 66 | 70 | 67.5 | 88 | 86.43 | 89.66 |
| Pro | 4 | 5 | 5.6 | 26 | 20 | 22.4 |
| Ser | 66.32 | 69.36 | 70.1 | 83.63 | 88.97 | 85 |
| Thr | 49.02 | 56 | 54.2 | 81.75 | 78.59 | 70 |
| Trp | 34 | 30.47 | 33.65 | 57.51 | 49.95 | 55 |
| Tyr | 68 | 66.76 | 69.1 | 90 | 82.61 | 85 |
| Val | 65 | 60.3 | 66.78 | 81 | 71.17 | 75.32 |

LC-MS data (For Fig. 2B)

0 h


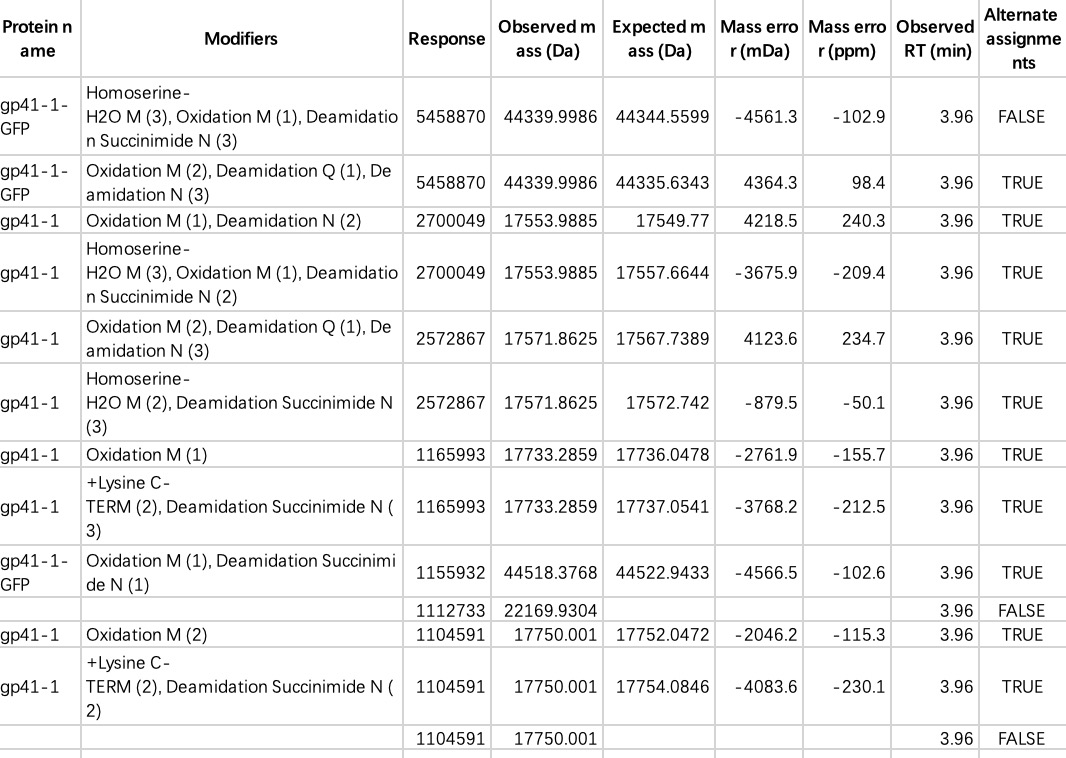


3 h


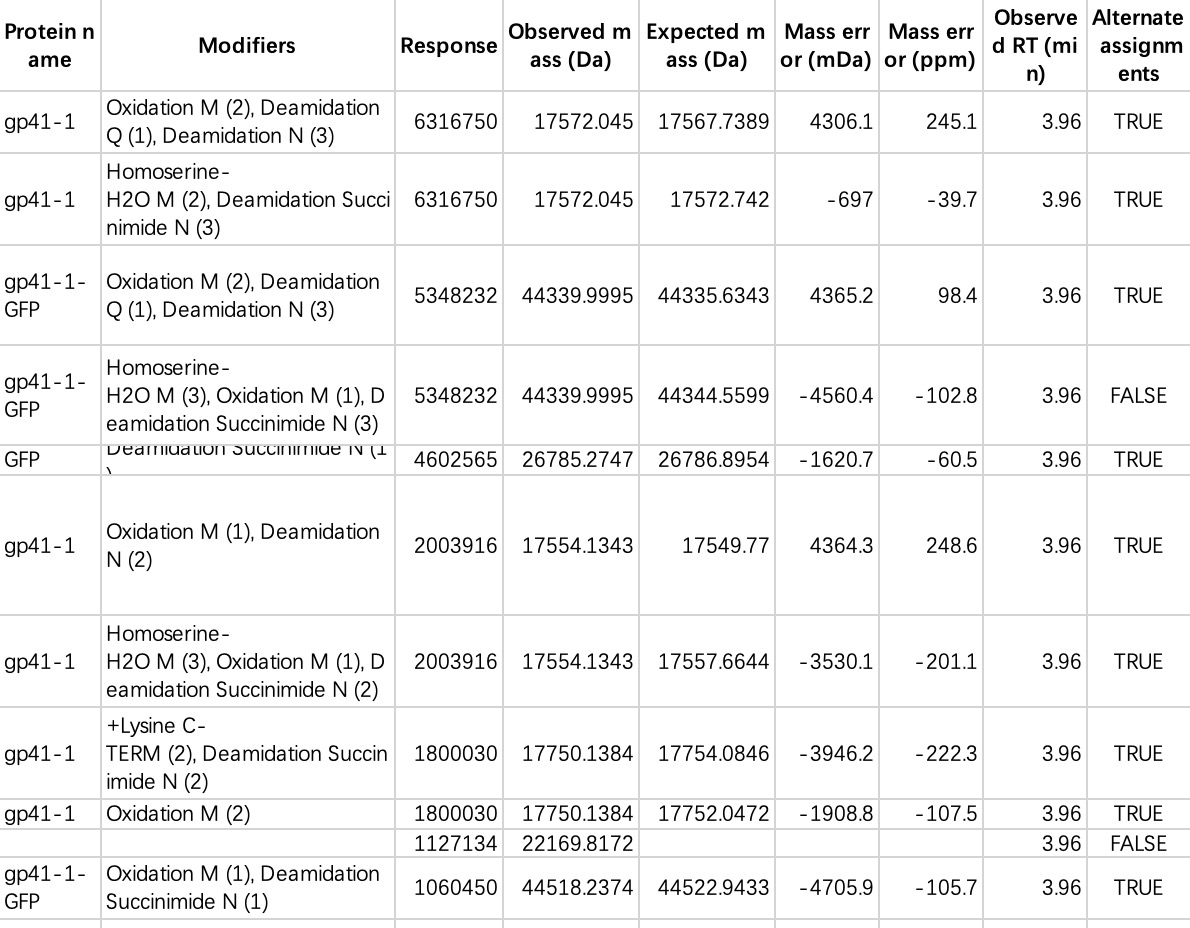


6 h


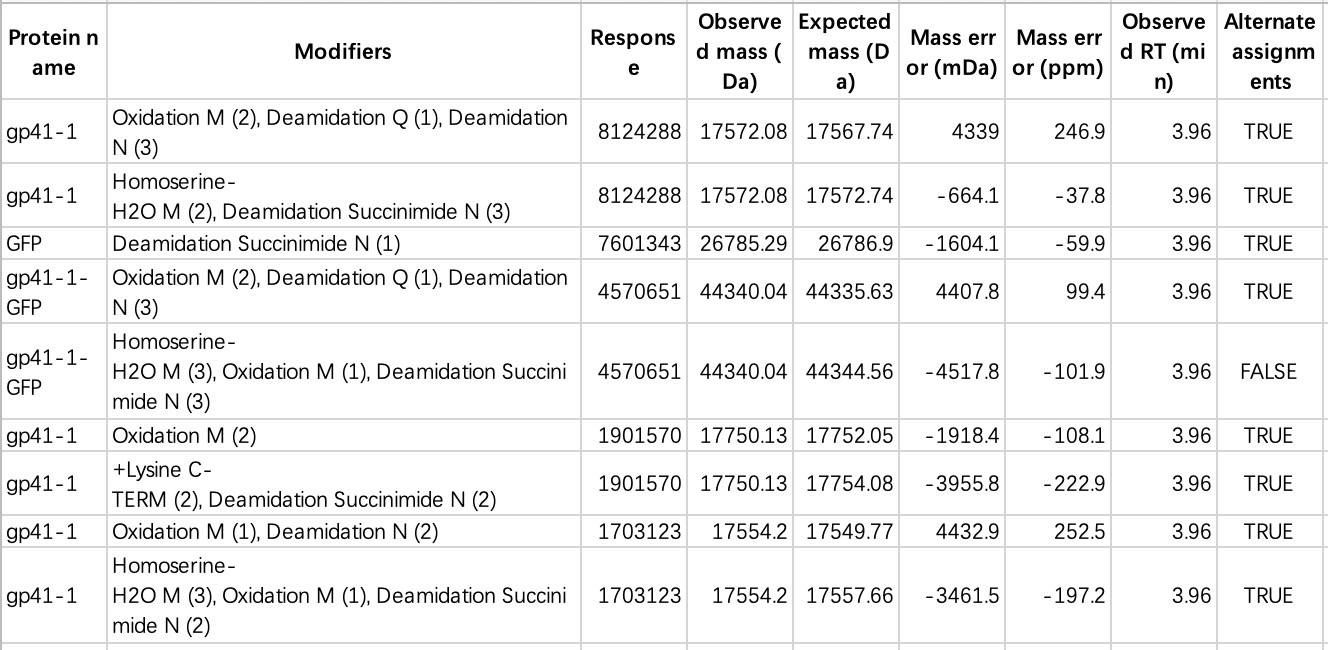


9 h


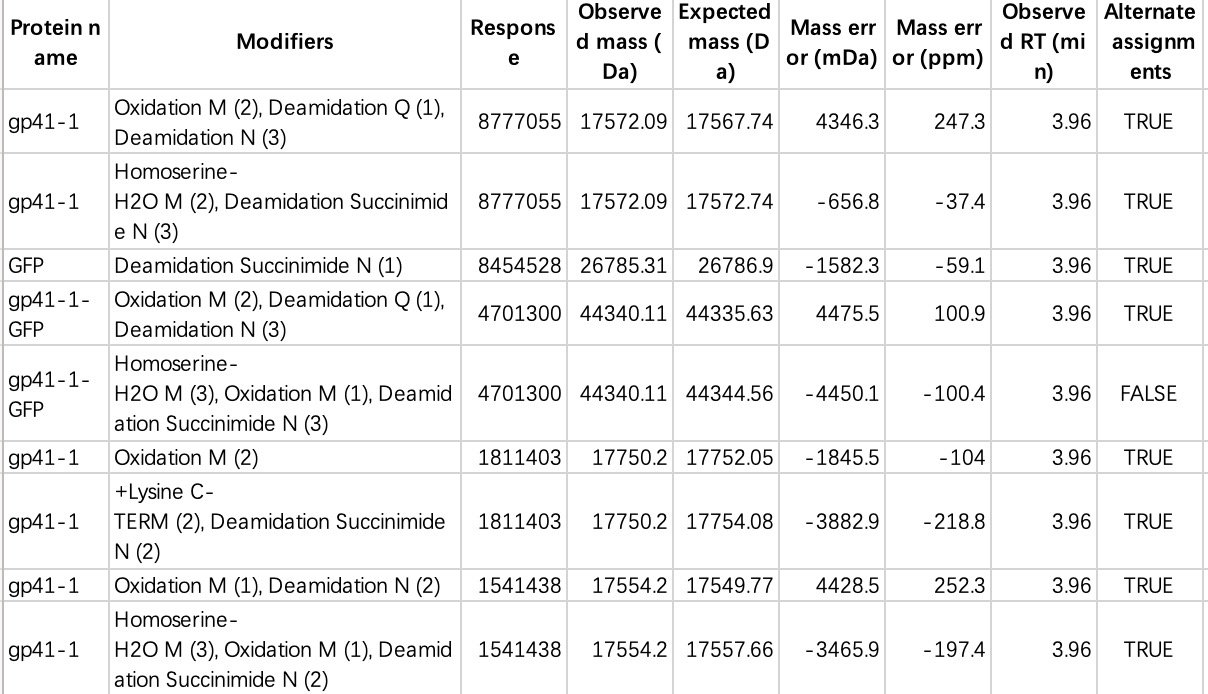


12 h


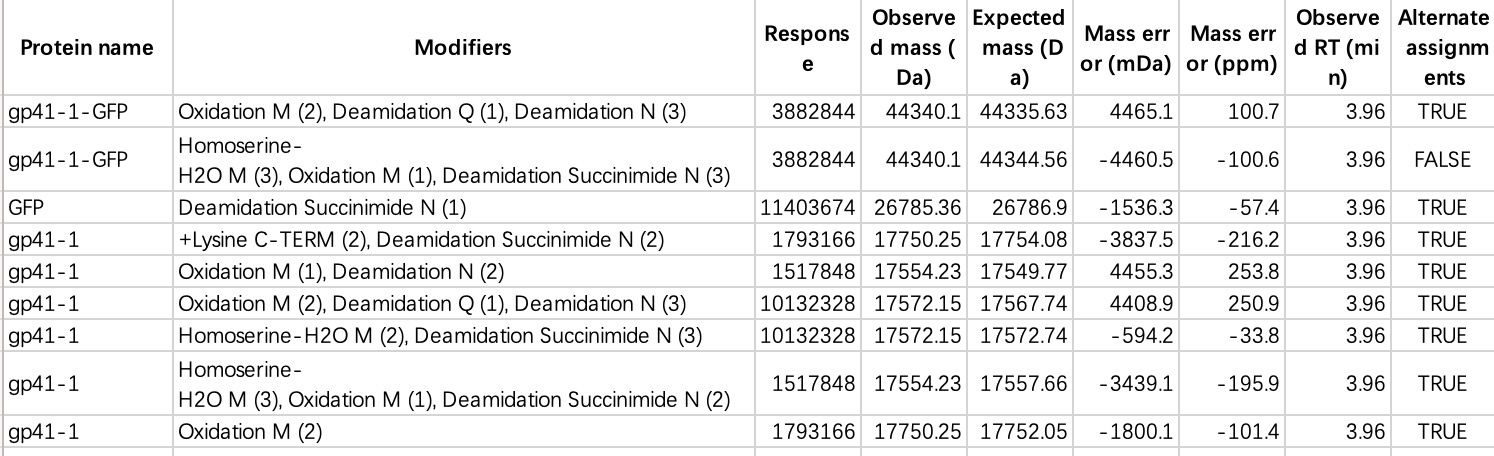


Cleavage under different pH, acetate (pH 4.4), Tris-HCl (pH 6, 8, 10) (For Fig. 4C)


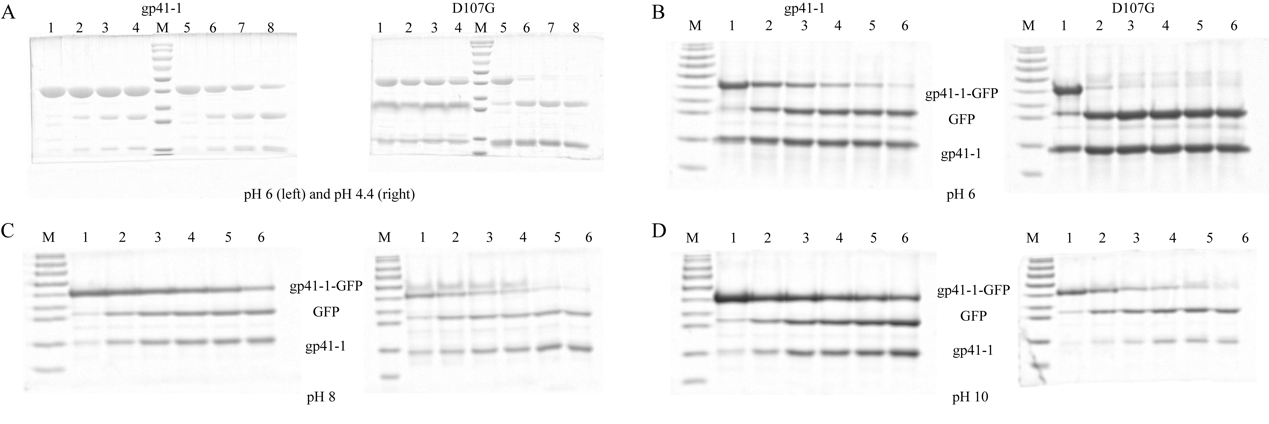


A) SDS-PAGE analysis of C-cleavage under Tris-HCl (pH6) and Acetate sodium (pH 4.4) for 3h. M: Marker, lane 1-4: cleavage Tris-HCl (pH6) for 0 h, 1 h, 2 h and 3 h. B) SDS-PAGE analysis of C-cleavage under Tris-HCl (pH6) for 24h. C) SDS-PAGE analysis of C-cleavage under Tris-HCl (pH8) for 24h. D) SDS-PAGE analysis of C-cleavage under Tris-HCl (pH10) for 24h. Lane 1-6: cleavage for 0 h, 3 h, 6 h, 9 h, 12 h and 24 h.

SDS-PAGE of T123Agp41-1 cleavage (For Fig. 4A and S7)


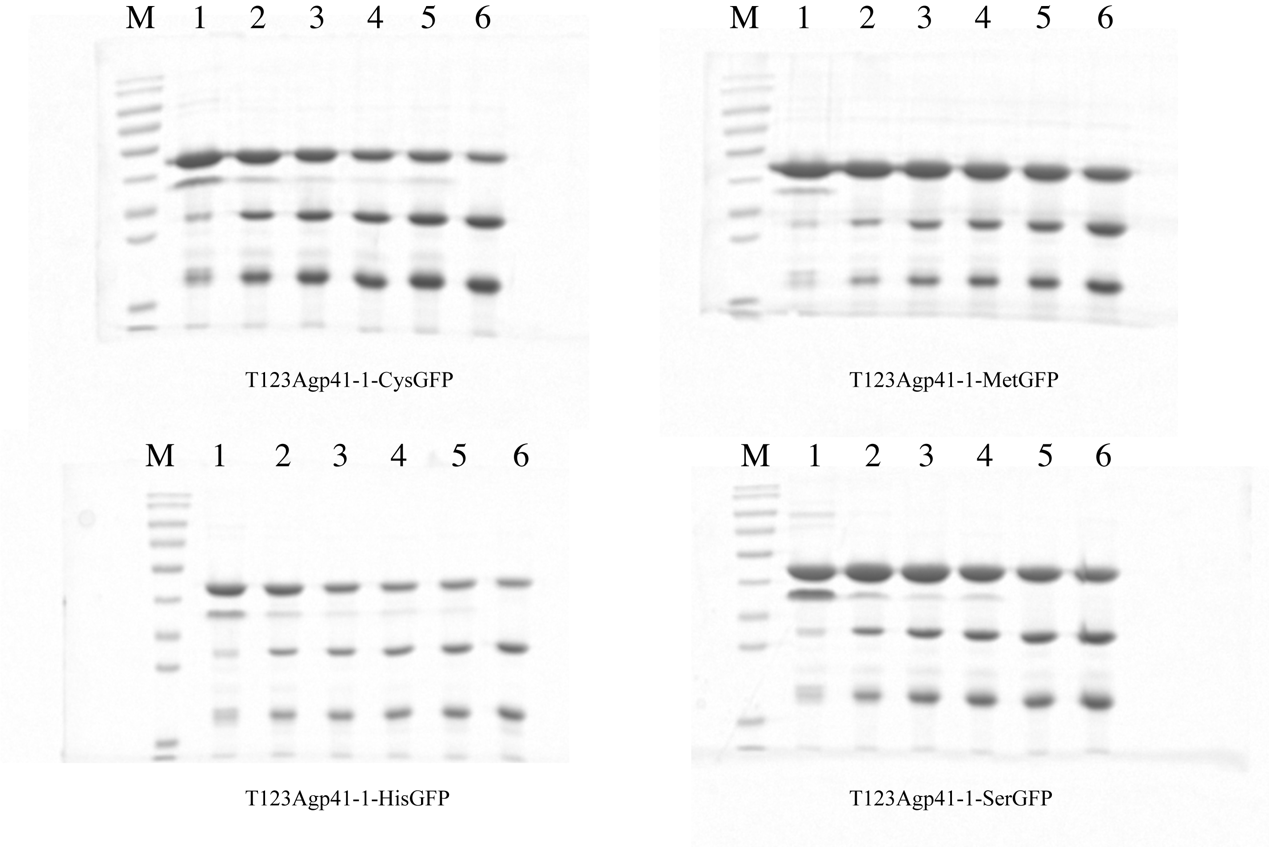


SDS-PAGE analysis of C-cleavage of T123Agp41-1 fused to different C+1 residues


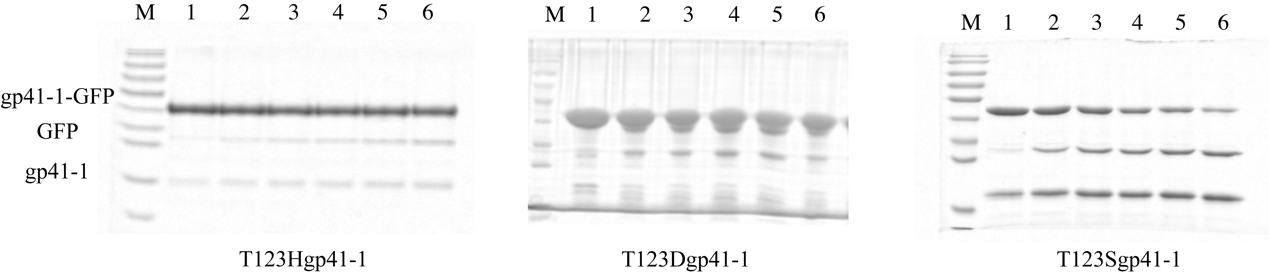


SDS-PAGE analysis of C-cleavage of T123H, T123D and T123S mutants

MD stimulations (For table 2)

χ2 angles of His


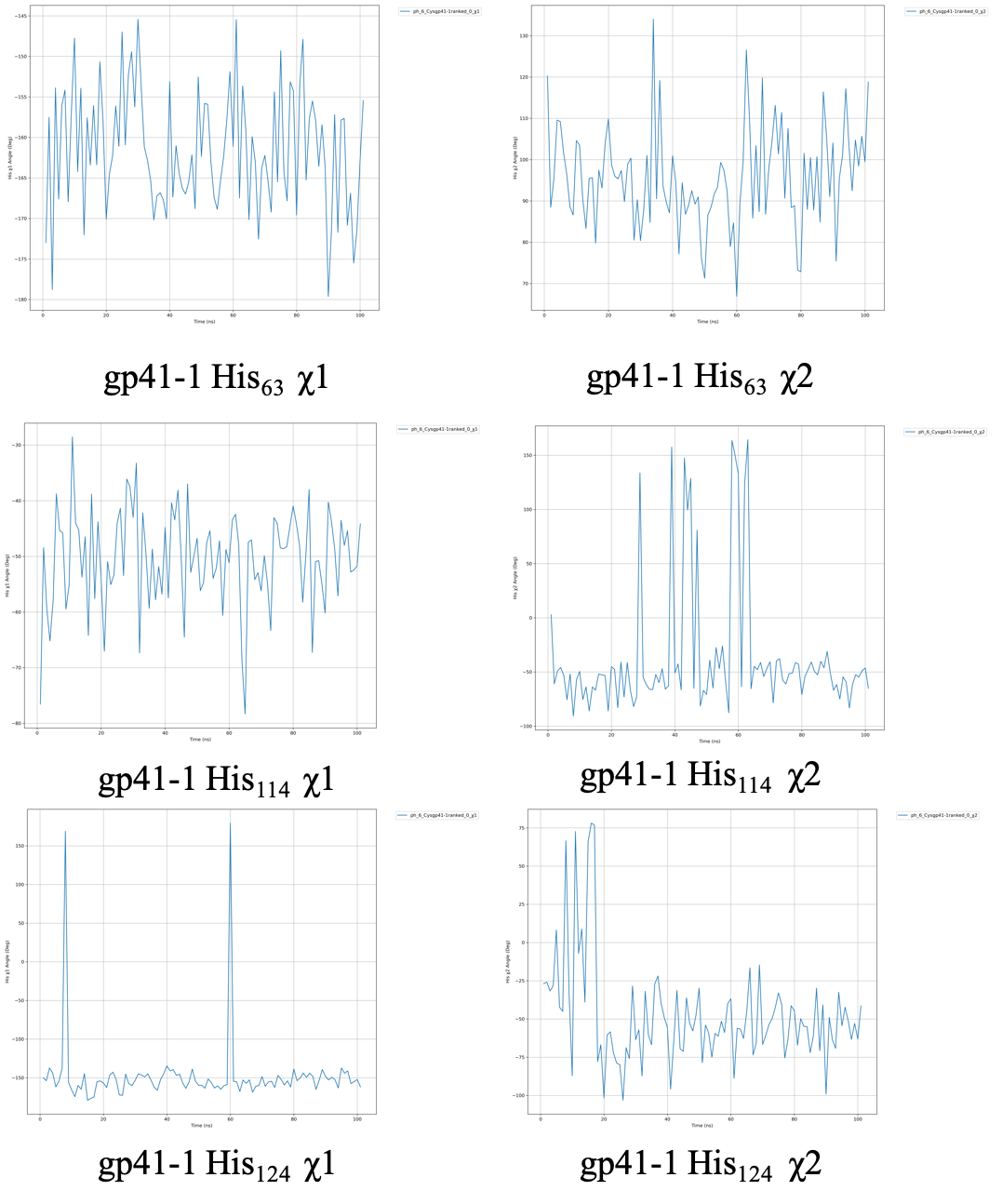


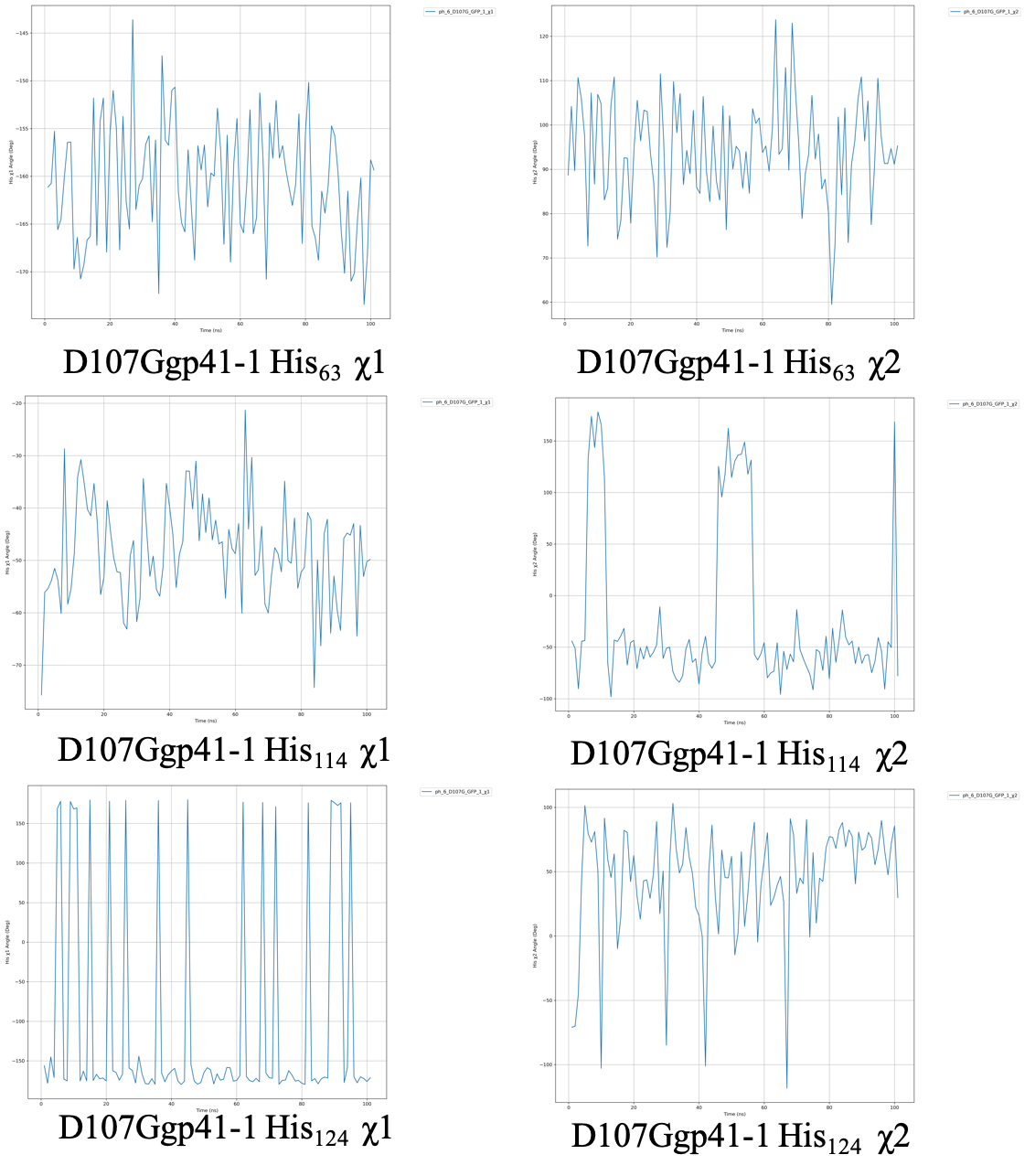


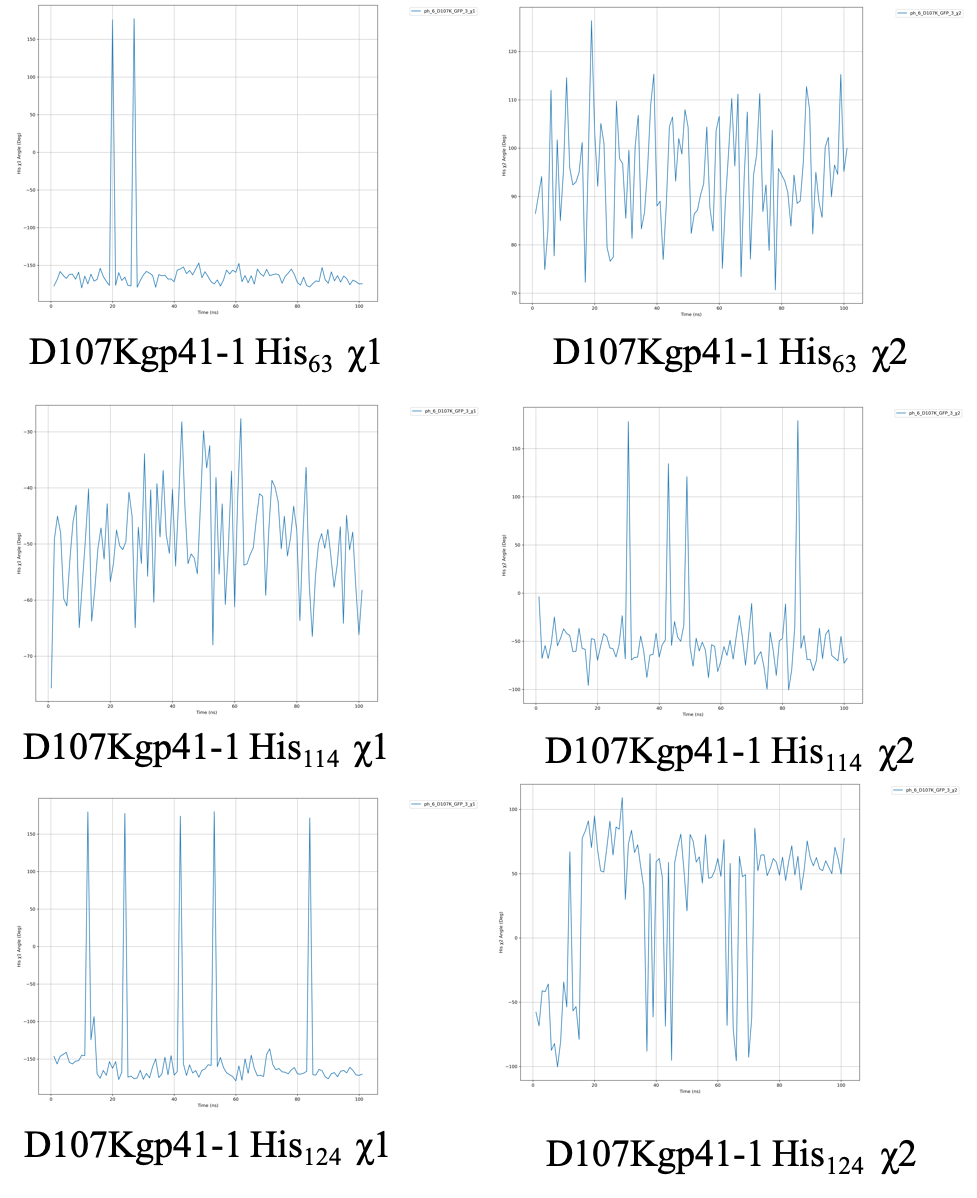


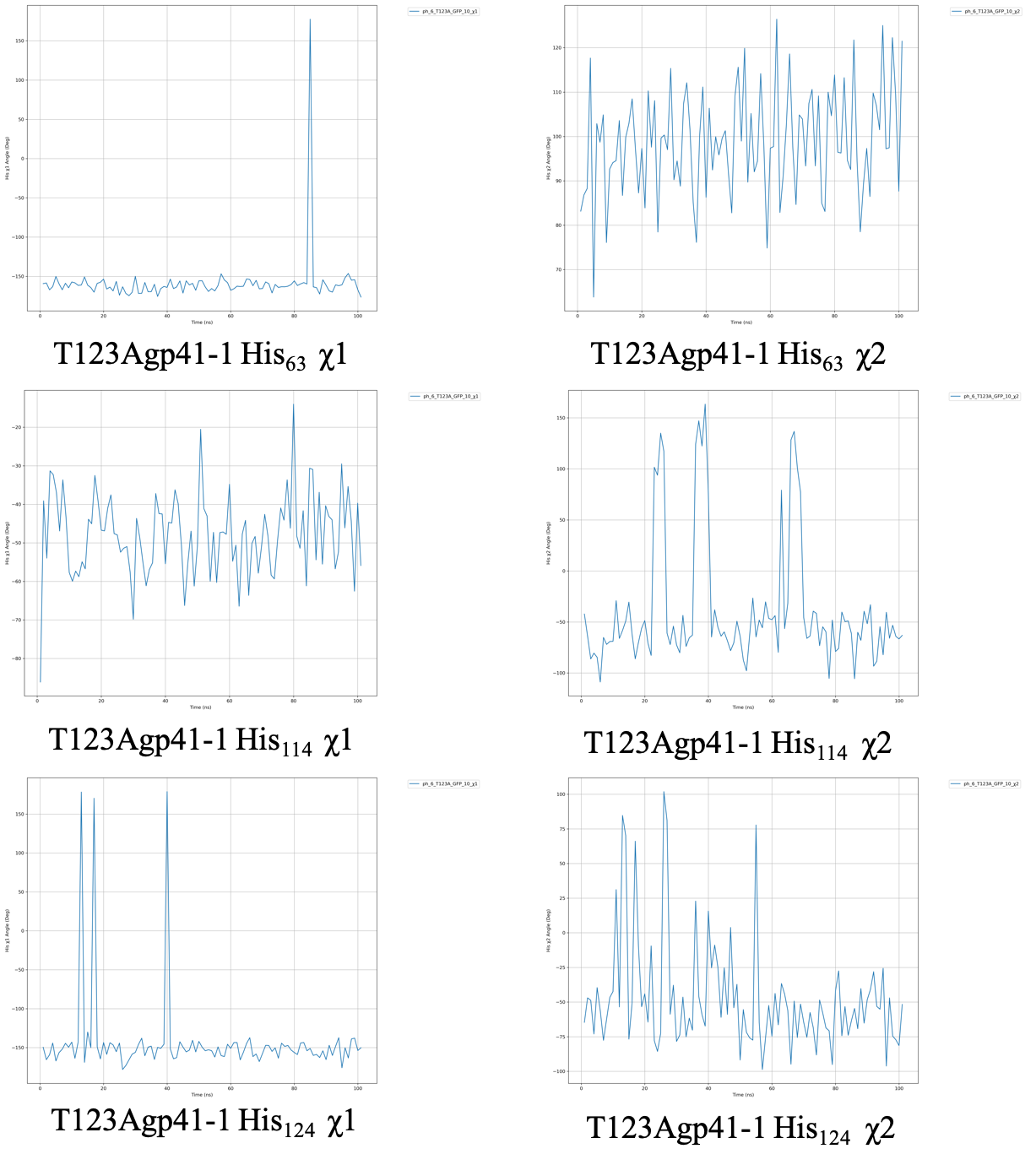


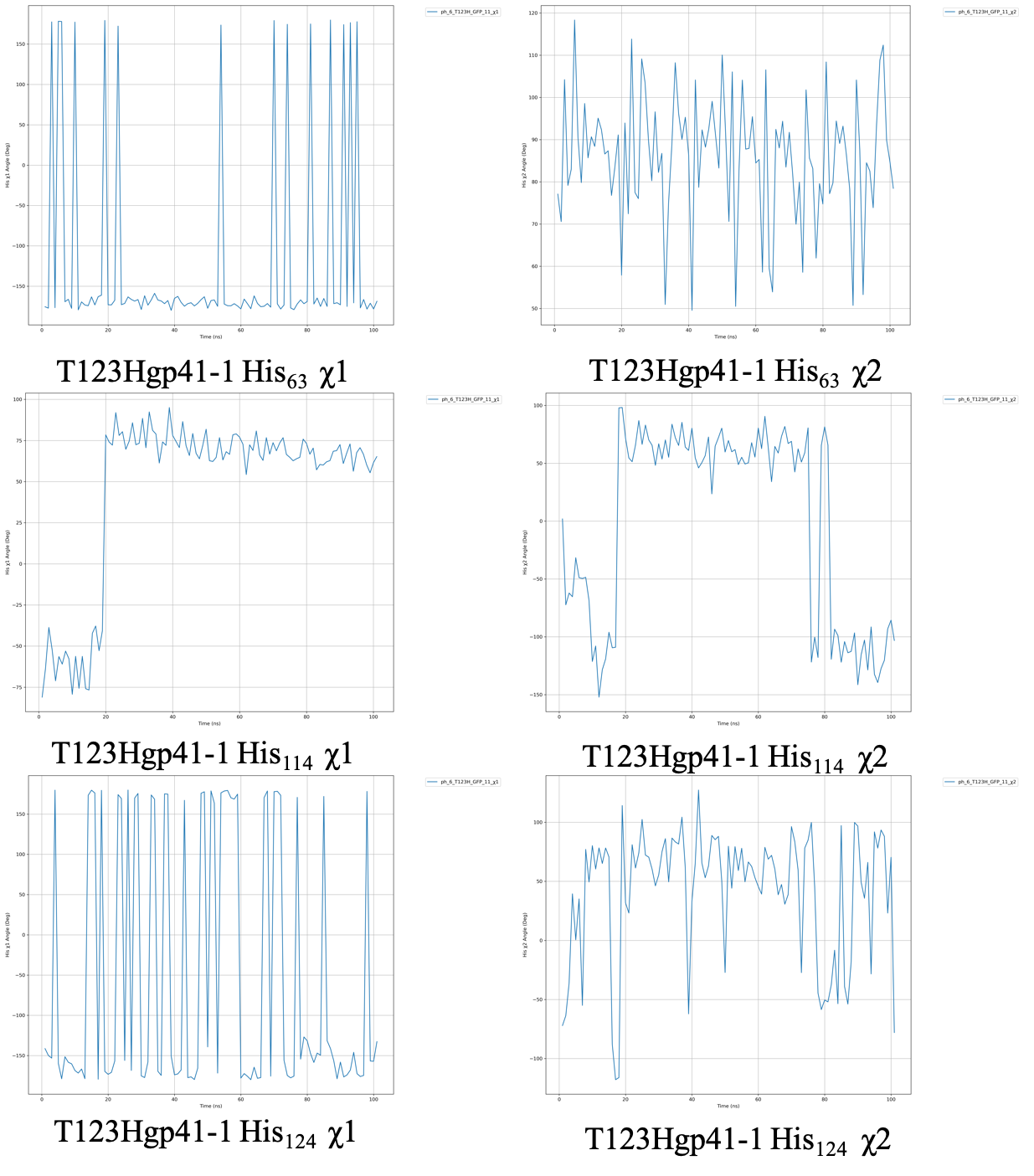


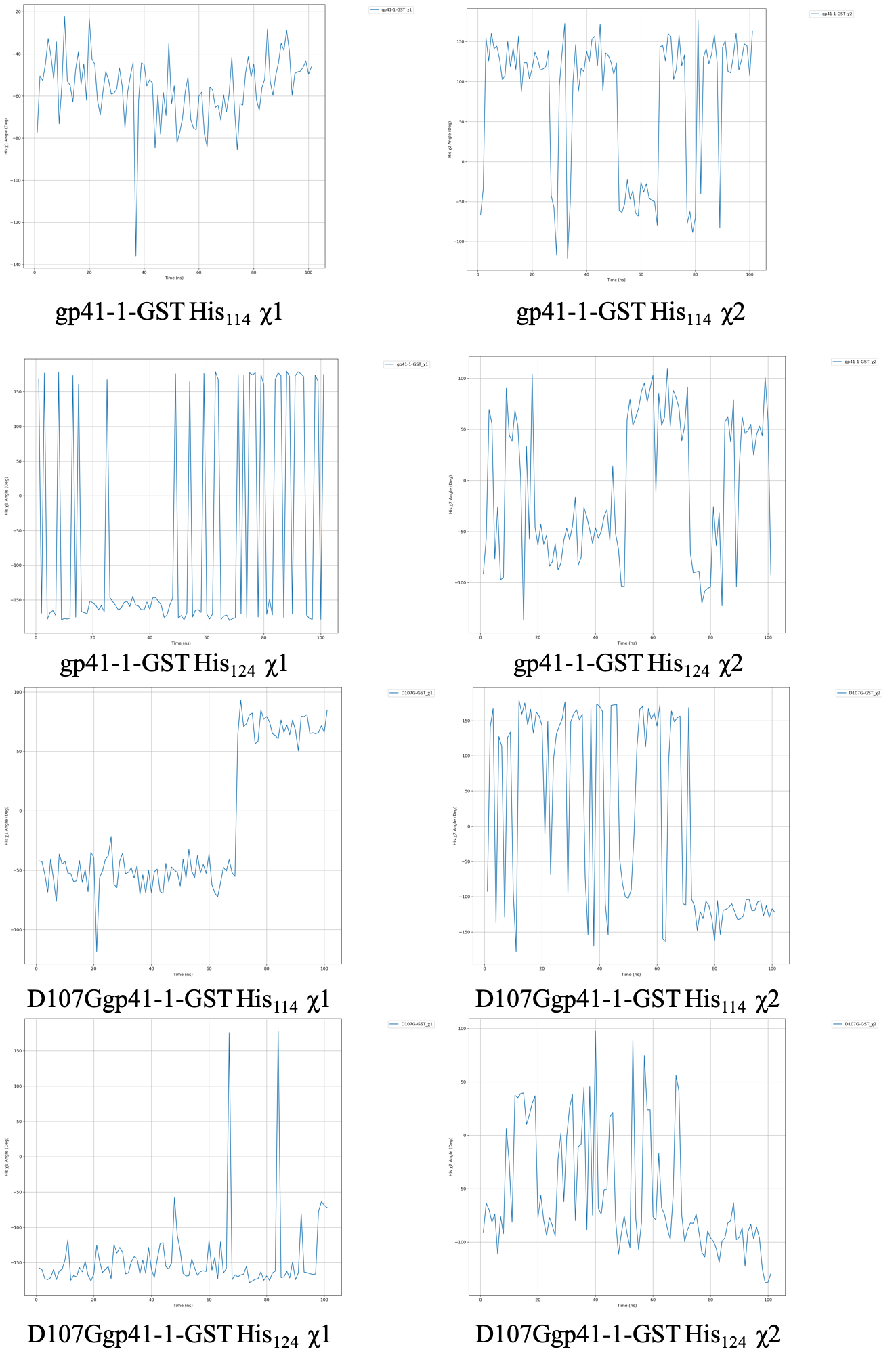


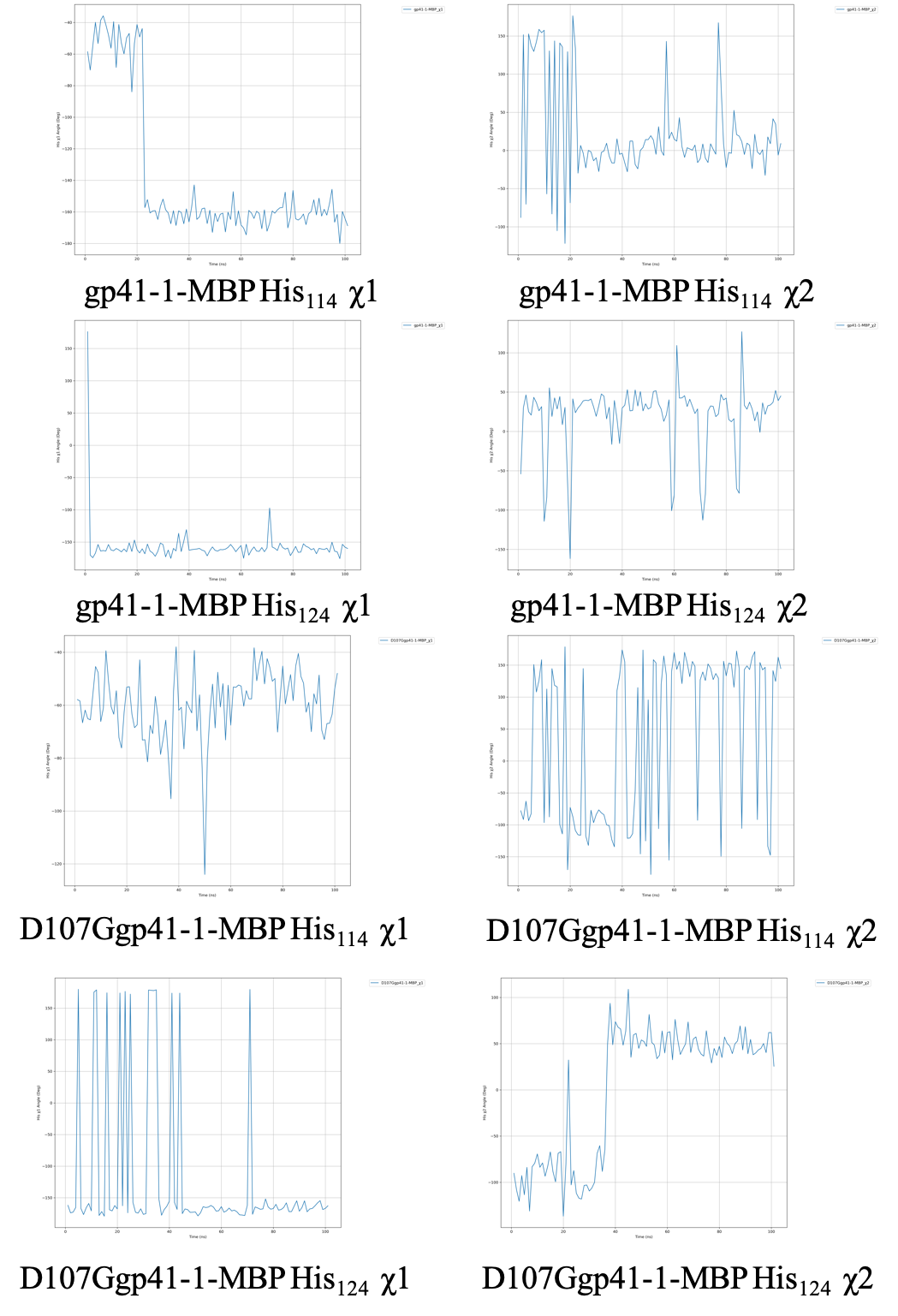


Distance between Asp_107_ and Asn_125_ (For Fig. 5)


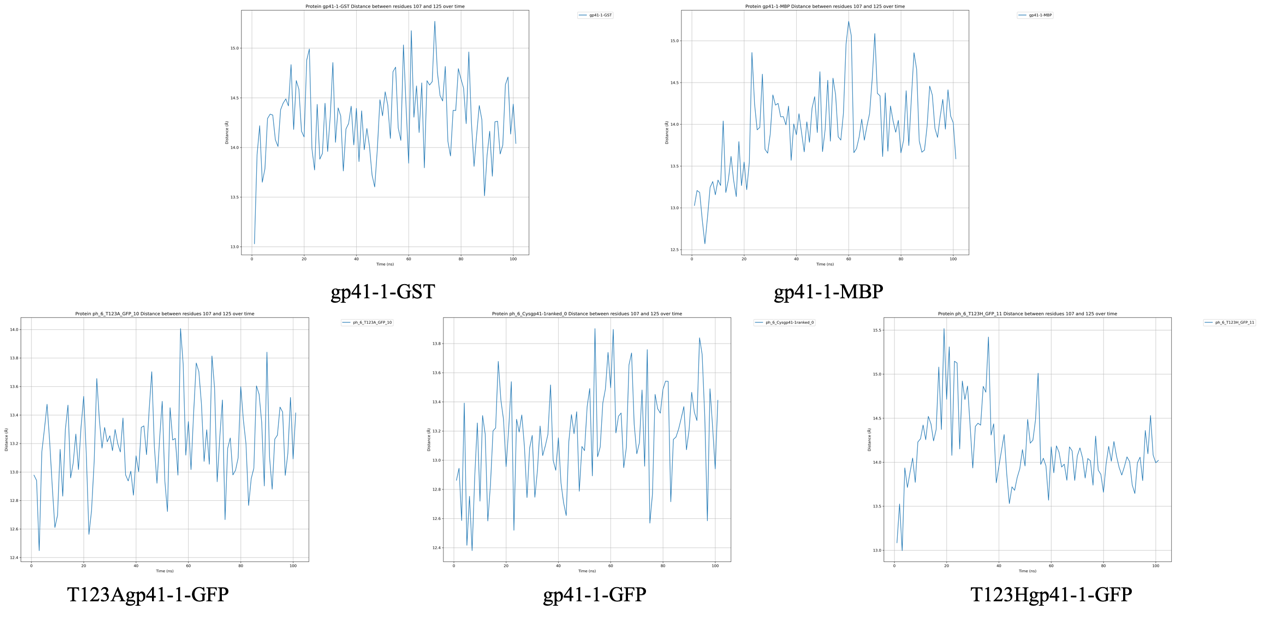

Supplement: Supplementary file 2 — Supporting Information [file ADVS-12-2501991-s002.docx]
